# Two Novel Endornaviruses Co-infecting a *Phytophthora* Pathogen of *Asparagus officinalis* Modulate the Developmental Stages and Fungicide Sensitivities of the Host Oomycete

**DOI:** 10.3389/fmicb.2021.633502

**Published:** 2021-02-03

**Authors:** Keiko Uchida, Kohei Sakuta, Aori Ito, Yumi Takahashi, Yukie Katayama, Tsutomu Omatsu, Tetsuya Mizutani, Tsutomu Arie, Ken Komatsu, Toshiyuki Fukuhara, Seiji Uematsu, Ryo Okada, Hiromitsu Moriyama

**Affiliations:** ^1^Laboratory of Molecular and Cellular Biology, Graduate School of Agriculture, Tokyo University of Agriculture and Technology, Fuchu, Japan; ^2^Research and Education Center for Prevention of Global Infectious Diseases of Animals, Tokyo University of Agriculture and Technology, Fuchu, Japan; ^3^Laboratory of Plant Pathology, Graduate School of Agriculture, Tokyo University of Agriculture and Technology, Fuchu, Japan

**Keywords:** asparagus phytophthora rot fungus, endornavirus, attenuation, fungicide sensitivity, zoosporangium formation, zoospore transmission

## Abstract

Two novel endornaviruses, Phytophthora endornavirus 2 (PEV2) and Phytophthora endornavirus 3 (PEV3) were found in isolates of a *Phytophthora* pathogen of asparagus collected in Japan. A molecular phylogenetic analysis indicated that PEV2 and PEV3 belong to the genus *Alphaendornavirus*. The PEV2 and PEV3 genomes consist of 14,345 and 13,810 bp, and they contain single open reading frames of 4,640 and 4,603 codons, respectively. Their polyproteins contain the conserved domains of an RNA helicase, a UDP-glycosyltransferase, and an RNA-dependent RNA polymerase, which are conserved in other alphaendornaviruses. PEV2 is closely related to Brown algae endornavirus 2, whereas PEV3 is closely related to Phytophthora endornavirus 1 (PEV1), which infects a *Phytophthora* sp. specific to Douglas fir. PEV2 and PEV3 were detected at high titers in two original *Phytophthora* sp. isolates, and we found a sub-isolate with low titers of the viruses during subculture. We used the high- and low-titer isolates to evaluate the effects of the viruses on the growth, development, and fungicide sensitivities of the *Phytophthora* sp. host. The high-titer isolates produced smaller mycelial colonies and much higher numbers of zoosporangia than the low-titer isolate. These results suggest that PEV2 and PEV3 inhibited hyphal growth and stimulated zoosporangium formation. The high-titer isolates were more sensitive than the low-titer isolate to the fungicides benthiavalicarb-isopropyl, famoxadone, and chlorothalonil. In contrast, the high-titer isolates displayed lower sensitivity to the fungicide metalaxyl (an inhibitor of RNA polymerase I) when compared with the low-titer isolate. These results indicate that persistent infection with PEV2 and PEV3 may potentially affect the fungicide sensitivities of the host oomycete.

## Introduction

Mycoviruses are widespread in all major fungal taxa in nature, and increasing numbers of mycoviruses are currently being identified through metatranscriptomics (Ghabrial and Suzuki, 2009; Pearson et al., 2009; Xie and Jiang, 2014; Ghabrial et al., 2015; Marzano et al., 2016). Mycoviruses are transmitted vertically via spores and, in most cases, horizontally via hyphal anastomosis (Ikeda et al., 2003; Tuomivirta et al., 2009; Ong et al., 2016). Most mycoviruses do not cause any visible abnormal symptoms in the fungal hosts. However, some mycoviruses cause phenotypic changes such as reduced pathogenicity and/or reduced growth rates (Nuss, 2005; Ghabrial et al., 2015). Such infections might be exploited in the biological control of fungal diseases.

The *Endornaviridae* family includes viruses with linear RNA genomes that range from 9.8 to 17.6 kb. Their genomes contain single open reading frames (ORFs) that encode polyproteins ranging from 3,217 to 5,825 amino acids (Valverde et al., 2019). Currently, this family is classified into two genera, *Alphaendornavirus* and *Betaendornavirus*, based on genome size, host type, and unique domains (Adams et al., 2017). Alphaendornaviruses infect plants, fungi, and oomycetes, while betaendornaviruses infect ascomycete fungi (Fukuhara et al., 2006; Valverde et al., 2019).

Most endornaviruses do not cause any apparent symptoms in their hosts. Indeed, endornaviruses have been widely detected in plant crops including broad bean (*Vicia faba*) (Grill and Garger, 1981; Pfeiffer, 1998), common bean (*Phaseolus vulgaris*) (Wakarchuk and Hamilton, 1985, 1990; Mackenzie et al., 1988; Okada et al., 2013), pepper (Valverde and Gutierrez, 2007), rice (Moriyama et al., 1995), and melon (*Cucumis melo*) (Coutts, 2005), and in most cases there were no obvious disease symptoms. There are also few reports on the effects of endornavirus infection on host growth in oomycetes and filamentous fungi (Hacker et al., 2005; Yang et al., 2018). However, some studies have revealed less obvious symptoms of endornavirus infection. For example, Vicia faba endornavirus causes cytoplasmic male sterility in *V. faba* (Grill and Garger, 1981; Turpen et al., 1988; Lefebvre et al., 1990; Pfeiffer et al., 1993), and Helicobasidium mompa endornavirus 1–670 decreases the virulence of the violet root rot fungus *Helicobasidium mompa* (Osaki et al., 2006). The first endornavirus detected in the genus *Phytophthora* was Phytophthora endornavirus 1 (PEV1), found in a *Phytophthora* sp. isolated from Douglas fir (Hacker et al., 2005). This endornavirus also has no noticeable impact on its host.

*Phytophthora* is a genus of plant pathogenic oomycetes that can infect a wide range of hosts including field crops, vegetable crops, fruit trees, ornamental plants, and tree plants all over the world. Many *Phytophthora* species are polyphagous and are important pathogens in agriculture and forestry. In 2014, 123 valid species were reported, and the numbers are still increasing. *Phytophthora* species can be divided into at least 10 clades based on the sequences of their rDNA ITS regions (Cooke et al., 2000; Blair et al., 2008). These oomycetes belong to the superphylum Stramenopiles, and are phylogenetically related to brown algae and diatoms (Cavalier-Smith and Chao, 2006; Webster and Weber, 2007).

*Phytophthora* rot of asparagus was first reported in California (Ark and Barret, 1938), and the pathogen was subsequently identified as *Phytophthora sojae* (Falloon, 1982). Asparagus has also been infected by *Phytophthora nicotianae* in Peru (Aragon-Caballero et al., 2008) and by *Phytophthora asparagi* in Michigan (Saude et al., 2008; Crous et al., 2012). In Japan, *P*. *nicotianae* infections of asparagus have been detected in Ehime and Saga prefectures, and a *Phytophthora* sp. has also been reported to infect asparagus in Toyama, Fukushima, and Hokkaido prefectures (Kodama et al., 2015). Control of these oomycete diseases relies mainly on fungicides from 16 different chemical groups including phenylamides, quinone outside inhibitors, carboxylic acid amides, and multisite inhibitors. However, the frequent use of fungicides can lead to fungicide-resistant oomycetes that are difficult to control chemically.

To date, there have been few reports on mycoviral infections of *Phytophthora* species. *Phytophthora infestans*, which is the causal agent of potato late blight, has been shown to host four unclassified double stranded RNA (dsRNA) viruses named Phytophthora infestans RNA viruses 1–4 (PiRV1–4) (Cai et al., 2009, 2012, 2013, 2019). The alphaendornavirus PEV1 was found in a *Phytophthora* isolate from Douglas fir in the United States (Hacker et al., 2005), and similar virus strains have since been found in *Phytophthora ramorum* isolates from various host plants including *Rhododendron* and *Viburnum* species in both the United States and Europe (Kozlakidis et al., 2010).

Previous studies of endornaviruses have mainly focused on their isolation, sequencing, and genome structures. Therefore, little is known about the effects of infection by endornaviruses on their hosts. In this study, we analyzed the genome organization and phylogeny of two novel endornaviruses (Phytophthora endornavirus 2, PEV2 and Phytophthora endornavirus 3, PEV3), which were discovered in a *Phytophthora* rot pathogen of asparagus. We identified *Phytophthora* host isolates with both high and low titers of the viruses, and used these to analyze the effects of PEV2 and PEV3 infection on the host phenotype and its sensitivity to several oomycete fungicides.

## Materials and Methods

### Pathogen Isolates and Culture Methods

We screened 68 *Phytophthora* and 2 *Pythium* isolates for infection with mycoviruses, by looking for the presence of dsRNA molecules in mycelial cells. Sixty-one of these isolates represented 7 *Phytophthora* species found in a variety of plants from multiple locations in Japan (Supplementary Table 1). The remaining 7 *Phytophthora* sp. isolates were collected from asparagus (*A. officinalis*) in Toyama, Hokkaido, Akita, and Fukushima prefectures (Table 1 and Supplementary Table 1). Among these, isolates CH98ASP060, CH98ASP059, and Ku-1 were collected from asparagus storage roots while isolates Ak-6-1 and Fk-3 were collected from rhizomes. The isolates were cultured on PDA (potato dextrose medium with 2% agar) at 25°C in the dark for 14 days, and then stored at 15°C on slants of modified Weitzman-Silva-Hunter agar (WSH; 10 g oatmeal, 1 g NaH_2_PO_4_, 1 g MgSO_4_⋅7H_2_O, 1 g KNO_3_, 20 g agar, 1,000 ml distilled water). For all experiments involving growth of the isolates, mycelia were precultured on PDA medium at 25°C in the dark for 7 days.

**TABLE 1:** Sources of the *Phytophthora* isolates used in the study, and presence of endornaviruses in each isolate.

**Isolate**	**Location**	**Infecting endornaviruses**	
	
		**dsRNA^a^**	**Contents^b^**
CH98ASP060	Toyama	PEV2&PEV3	+++
CH98ASP060-a	Toyama	PEV2&*PEV*3#	++
CH98ASP051	Toyama	PEV2&PEV3	+++
CH98ASP059	Toyama	PEV2&PEV3	+++
CH98ASP059-L	Toyama	PEV2#&*PEV*3#	+
CH98ASP066	Toyama	PEV2&PEV3	+++
Ku-1	Hokkaido	14-kb dsRNAs	+++++
Ak-6-1	Akita	PEV2&PEV3	++
Fk-3	Fukushima	PEV2&PEV3	+++

*^*a*^# Invisible by EtBr staining (0.5 mg/ml) but detected by RT-PCR. PEV2; 14.3 kb, PEV3;13.8 kb. ^*b*^dsRNA contents were estimated as described in the Section “Materials and Methods.”*

### Extraction and Analysis of dsRNAs by Electrophoresis and RT-PCR

Mycelial discs (4 mm diameter) were removed from fungal mats grown on PDA using a cork borer. The disks were used to inoculate 1/3 YG liquid medium (0.17% yeast extract, 0.67% glucose), with 3 disks in 25 ml cultures or 20 disks in I L cultures. The cultures were incubated at 26°C for 2 weeks in the dark on a reciprocal shaker with 60 oscillation per min, then filtered through gauze. The mycelia were dried in a commercial dryer (SIS Co., Ltd., Japan) at 65°C for 10 min, and then stored at −80°C until used for dsRNA extraction. The mycelial yields were about 0.2 g from the 25 ml cultures and about 1 g from the 1 L cultures.

We used micro-spin columns with Cellulose D (Advantec, Japan) to extract total nucleic acids and then purify dsRNAs as described by Okada et al. (2015). Fungal mycelium (0.1 g dry weight) was pulverized in 500 μl extraction buffer [100 mM NaCl, 10 mM Tris-HCl pH 8.0, 1 mM EDTA, 1% SDS, and 0.1% (v/v) β-mercaptoethanol) and then extracted twice with equal volumes of phenol-chloroform-isoamyl alcohol (25:24:1). The aqueous phase was mixed with ethanol (final concentration 16%), and the dsRNA was purified using a spin column as described by Okada et al. (2015). Finally, the dsRNAs were concentrated by ethanol precipitation and stored at −30°C.

The purified dsRNAs were visualized by electrophoresis in 0.8% or 0.6% agarose gels stained with ethidium bromide. The level of dsRNA in each isolate was estimated using a gel imaging system (Ez-Capture MG ATTO, Japan). These estimates are indicated by + signs in Table 1.

Reverse transcription (RT)-PCR with virus-specific primers was also used to detect the PEV2 and PEV3 dsRNAs. We used the primers shown in Supplementary Table 2 and set up the reactions using the SuperScript III One-Step RT-PCR System with Platinum Taq, following the manufacturer’s protocol (Life Technologies, Carlsbad, CA, United States). The thermal cycling conditions were as described previously, with 40 cycles of amplification and an annealing temperature of 55°C (Komatsu et al., 2016).

### Cloning and Sequence Analyses

Double-stranded RNAs extracted from the original isolate CH98ASP060 were used as templates for cDNA synthesis, and a series of overlapping cDNA clones were obtained as described by Aoki et al. (2009). These cDNA clones were sequenced using an Applied Biosystems 3130xl Genetic Analyzer. In addition, dsRNA purified from isolate CH98ASP060 was sequenced by next-generation sequencing using the Miseq system (Illumina Co., Ltd.; Miseq System Catalog No. MS-J-001). We obtained 2,270,543 raw reads that were assembled into 2,255 contigs, of which 28 contigs had coverage of more than 100. The 5′- and 3′-terminal sequences of each dsRNA segment were determined using the SMARTer^®^ RACE cDNA Amplification Kit (Clontech Laboratories, Inc., Mountain View, CA, United States) (Frohman et al., 1988). We followed the manufacturer’s protocol, and used the primers shown in Supplementary Table 2.

The nucleotide sequences were analyzed for ORFs, and the ORFs were translated into amino acid sequences using GENETYX version 9 software (GENETYX, Japan). We also used GENETYX to perform protein similarity searches. Multiple alignments based on the putative amino acid sequences were obtained by performing a series of pairwise alignments using CLUSTAL_X version 2.0 (Thompson et al., 1997; Larkin et al., 2007) and MEGA6 software (Tamura et al., 2013). Phylogenetic analysis under the maximum likelihood framework was performed using the optimal model of amino acid substitutions selected by ProtTest 2.4 and PhyML3.1 (Guindon et al., 2010). The bootstrap test was performed with 1,000 re-samplings.

### Measurement of Hyphal Growth, Induction of Zoosporangium Formation, and Assessment of Virus Transmission via Monozoospores

To measure hyphal growth, mycelial disks (4 mm diameter) were cut from the margins of precultured colonies, transferred to fresh PDA plates, and incubated at 25°C in the dark for 7 days. For each isolate, average values for colony diameter were determined by measuring three independent colonies.

To induce zoosporangium formation, sterilized 1 cm × 1 cm filter paper pieces (Whatman 3MM) were placed on fresh PDA medium supplemented with 25 μg/ml β-sitosterol (MP Biomedicals, LLC, United States), and two precultured mycelial disks were placed adjacent to each piece of paper. These were incubated at 25°C in the dark for 14 days (Hendrix, 1970). Three pieces of filter paper covered with mycelia were then transferred to 50 ml of sterile rainwater in a flask, and cultured at 18°C under full-light conditions for 40 h with gentle shaking. The formation of zoosporangia and release of zoospores were confirmed under an optical microscope (Olympus IX71; Tokyo, Japan), and the zoospore density was measured using a hemocytometer (Thoma). For each isolate, the average rate of zoosporangium formation was determined by counting the numbers of zoosporangia formed at the edges of 9 filter paper pieces (*n* = 9).

To assess virus transmission in the monozoospores, the zoospore concentrations were adjusted to 1 × 10^4^ zoospores/ml, and 200 μl of each zoospore suspension was spread on PDA medium and cultured at 25°C in the dark for 2 days. Germinating hyphae from the monozoospores were transferred to flasks containing 50 ml of PD broth and incubated at 25°C in the dark for 14 days with gentle shaking. The dsRNAs were extracted from the resulting mycelia and visualized by gel electrophoresis as described above. RT-PCR was also performed using specific primers to detect PEV2 and PEV3.

### Cystospore Formation and Determination of Cystospore Germination Rates

After zoosporangium formation was induced as described above, cystospore formation was induced using the vibration method with some modifications (Tokunaga and Bartnicki-Garcia, 1971; Kliejunas and Ko, 1974; Ko and Chan, 1974). The filter paper pieces with zoosporangia were moved from 18 to 25°C and incubated for 0.5 to 1 h to promote indirect germination of the zoospores (i.e., release of motile zoospores from the zoosporangia). We confirmed that the efficiency of indirect germination was more than 50%, which is the accepted diagnostic check for biologically functional zoosporangia. To induce encystment of the zoospores, 0.5 ml of the zoospore suspension was transferred to a 1.5 ml microtube and subjected to vibration at 1,800 rpm for 1–2 min (Deep Wellmaximizen, Bio shaker M., BR-022up, TITEC, Japan). The concentration of cystospores was determined using a hemocytometer and then adjusted to about 1 × 10^4^ cystospores/ml. An aliquot (200 μl) of the suspension was spread on a PDA plate and incubated for 2 days at 25°C. The visible hyphal colonies were counted under a stereomicroscope, and the rates of cystospore hyphal germination were calculated.

### Fungicide Sensitivity Tests

We used the following four fungicides in the sensitivity tests: benthiavalicarb-isopropyl (98.0%; FUJIFILM Wako Pure Chemical Corp.), famoxadone (98.8%; FUJIFILM Wako Pure Chemical Corp.), metalaxyl (98%; Toronto Research Chemicals, Inc.), and chlorothalonil (99.9%; FUJIFILM Wako Pure Chemical Corp.). Each chemical was dissolved in acetone (10 mg ml^–1^) and stored at 5°C in the dark until used. n-Propyl gallate (PG; MP Biomedicals, Inc.) was used with the quinone outside inhibitor fungicide famoxadone to inhibit the activity of cyanide-insensitive alternative oxidase (AOX) (Hollomon et al., 2005; Ishii et al., 2009). The PG was dissolved in dimethyl sulfoxide to prepare a stock solution (1 M).

Mycelial disks (4 mm diameter) were cut from the margins of precultured colonies and transferred to fresh PDA media containing various concentrations of each fungicide. The final concentrations of the fungicides were as follows: 0, 0.003, 0.03, 0.3, 3, 30, and 150 μg ml^–1^ for benthiavalicarb-isopropyl; 0, 0.0015, 0.015, 0.15, 1.5, 15, 150, and 300 μg ml^–1^ for famoxadone; 0, 0.001, 0.01, 0.1, 1, 10, 100, and 500 μg ml^–1^ for metalaxyl; and 0, 0.004, 0.04, 0.4, 4, 40, 400, and 800 μg ml^–1^ for chlorothalonil. The media containing famoxadone, including the 0 μg ml^–1^ famoxadone plates, also contained 1 mM PG. Each fungicide was added aseptically to the molten PDA medium after sterilization at 120°C for 15 min. Colony diameters were measured after incubating for 14 days at 25°C. The rates of inhibition of mycelial growth were calculated as follows.

(1) $$Inhibition\left(\%\right)=\frac{\begin{array}{c} 1-(colonydiameteronfungicide-\\ amendedPDA-4mm) \end{array}}{\begin{array}{c} C\,o\,l\,o\,n\,y\,d\,i\,a\,m\,e\,t\,e\,r\,o\,n\,u\,n\,a\,m\,e\,n\,d\,e\,d\\ P\,D\,A-4\,m\,m \end{array}}\times 100$$

The mean rates were based on measurements of 3 colonies for each fungicide concentration. The minimum inhibitory concentration (MIC) for each fungicide was determined as the minimum concentration that would result in 100% inhibition of hyphal growth.

## Results

### Screening for Viral dsRNAs in Asparagus Phytophthora Rot

**FIGURE 1:**
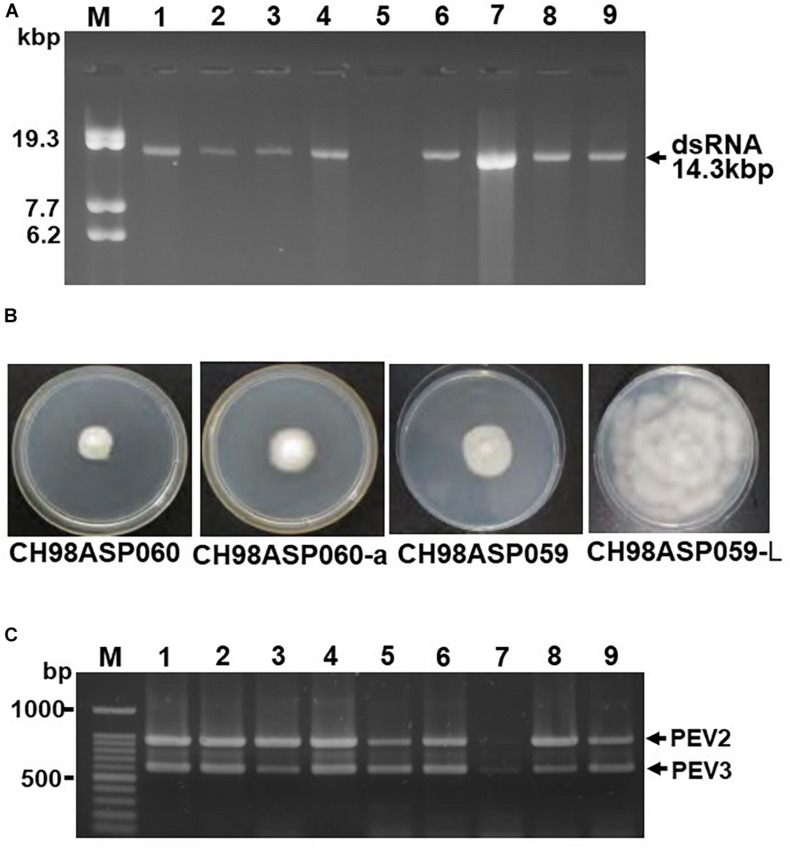
Detection of the dsRNA genomes of PEV2 and PEV3 in *Phytophthora* sp. isolates. **(A)** Agarose gel electrophoresis of dsRNAs purified from each of the isolates. Lane designation: M, DNA marker (250 ng of λ DNA digested with EcoT14I); 1, CH98ASP060; 2, CH98ASP060-a; 3, CH98ASP051; 4, CH98ASP059; 5, CH98ASP059-L; 6, CH98ASP066; 7, Ku-1; 8, Ak-6-1; and 9, Fk-3. The dsRNAs derived from each of the isolates (0.1 g dry weight) were electrophoresed on a 0.8% agarose gel for 18 h at 20 V and stained with ethidium bromide (0.5 μg/ml). Arrows indicate the positions of the 14.3 kb dsRNAs. **(B)** Hyphal morphologies of four isolates. The original isolate CH98ASP060 and its derivative, CH98ASP060-a, the original isolate CH98ASP059 and its derivative CH98ASP059-L were grown on PDA plates at 25°C for 14 days. **(C)** Results of one-step duplex RT-PCR amplification using PEV2 and PEV3-specific primers (Supplementary Table 2). Lane designation: M, DNA size markers; 1, CH98ASP060; 2, CH98ASP060-a; 3, CH98ASP051; 4, CH98ASP059; 5, CH98ASP059-L; 6, CH98ASP066; 7, Ku-1; 8, Ak-6-1; and 9, Fk-3.

We screened 68 *Phytophthora* isolates and 2 *Pythium* isolates for the presence of viral dsRNAs. Sixty-one of the *Phytophthora* isolates represented 7 known species (*P*. *nicotianae*, *Phytophthora cactorum*, *Phytophthora citrophthora*, *Phytophthora cryptogea*, *Phytophthora palmivora*, *Phytophthora citricola*, and *Phytophthora cinnamomi*) that were found in a variety of plants including fruit trees, ornamentals, and vegetable crops, collected from multiple locations in Japan (Supplementary Table 1). The remaining 7 *Phytophthora* sp. isolates have not yet been classified. They were collected from asparagus (*A. officinalis*) displaying phytophthora rot disease (Table 1 and Supplementary Table 1). We did not detect dsRNAs in any isolate except the 7 *Phytophthora* sp. isolates from asparagus. Each of these contained high molecular weight dsRNAs of approximately 14 kb, which were visually detected by ethidium bromide staining (Table 1 and Figure 1A). Four of these dsRNA-infected isolates, CH98ASP051, CH98ASP059, CH98ASP060, and CH98ASP066 (Figure 1A, lanes 1, 3, 4, and 6), were collected in Toyama prefecture in central Japan near the Sea of Japan coast. The other three isolates, Ku-1, Ak-6-1, and Fk-3 (Figure 1A, lanes 7, 8, and 9) were collected in Hokkaido, Akita, and Fukushima prefectures, respectively.

During the subculture of isolate CH98ASP060 on PDA medium, we noticed several sectors of mycelium with an altered phenotype. We subcultured the two phenotypes separately to create the sub-isolate CH98ASP060-a. The original isolate, CH98ASP060, displayed severely impaired hyphal growth, while CH98ASP060-a showed partially recovered hyphal growth (Figure 1B). The 14 kb dsRNA was also detected in CH98ASP060-a (Figure 1A, lane 2). After electrophoresis for an extended period in a 0.6% agarose gel, we realized that the original CH98ASP060 had two closely sized dsRNAs of approximately 13.8 and 14.3 kb (Supplementary Figure 1). In sub-isolate CH98ASP060-a, the 14.3 kb dsRNA band was clearly visible whereas the 13.8 kb band was only faintly visible (Supplementary Figure 1).

As described in more detail below, we designated the 14.3 kb dsRNA as PEV2 and the 13.8 kb dsRNA as PEV3, and created PCR primers specific for each of these dsRNAs (Supplementary Table 2). We performed duplex RT-PCR using both sets of primers and found that all but one of the *Phytophthora* sp. isolates from asparagus carried easily-detected levels of both the PEV2 and PEV3 dsRNAs (Table 1 and Figure 1C). These included the sub-isolate CH98ASP060-a (Figure 1C, lane 2). The isolate Ku-1 showed only faint amplification products (Figure 1C, lane 7), suggesting that this isolate may carry viral sequences that are similar, but not identical, to PEV2 and PEV3.

We also observed mycelium sectoring in the isolate CH98ASP059, and separated the sectors to create the sub-isolate CH98ASP059-L. The original isolate (CH98ASP059) showed impaired hyphal growth while the sub-isolate (CH98ASP059-L) displayed vigorous hyphal growth (Figure 1B). The dsRNA bands of approximately 14 kb were not detectable by gel electrophoresis of RNA isolated from CH98ASP059-L (Figure 1A, lane 5). However, when we performed duplex RT-PCR using the primers specific for PEV2 and PEV3, both dsRNAs were detected in CH98ASP059-L (Table 1 and Figure 1C, lane 5). Thus, we found that isolates containing high titers of the 14 kb dsRNAs showed attenuated growth (CH98ASP060, CH98ASP060-a, and CH98ASP059; Figure 1B) while the low-titer isolate (CH98ASP059-L) showed vigorous hyphal growth. This suggested that the attenuated growth may be caused by high levels of the PEV2 and PEV3 viruses in the hyphal cells.

### Nucleotide Sequence Analysis of PEV2 and PEV3

**FIGURE 2:**
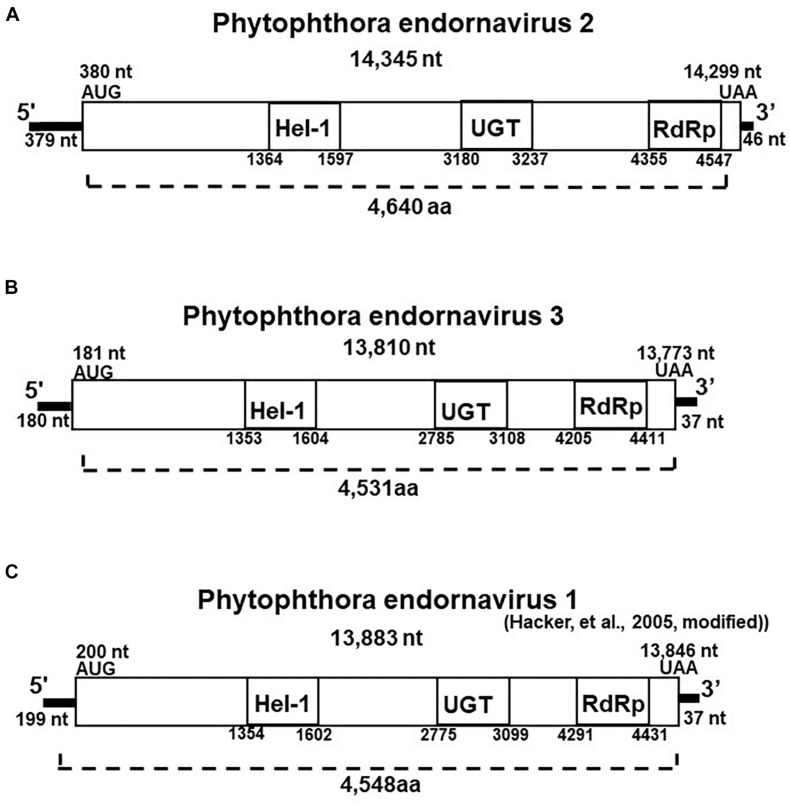
Properties of the Phytophthora endornavirus (PEV) genomes. The total nucleotide length of each dsRNA genomes is shown below the virus name. The predicted amino acid numbers are shown. The boxes represent the large ORFs, whereas lines depict UTRs. Hel-1, viral helicase 1; UGT, UDP-glycosyltransferase; RdRp, viral RNA dependent RNA polymerase. **(A)** Genome organization of PEV2. The viral genome is 14,345 nt in length. **(B)** Genome organization of PEV3. The viral genome is 13,810 nt in length. **(C)** Genome organization of PEV1. The viral genome is 13,883 nt in length.

We created cDNA clones and sequenced the 14.3 and 13.8 kb viral dsRNAs from isolate CH98ASP060. Based on their genome size, genome organization, and similarity to other endornaviruses, we tentatively designated the 14.3 kb dsRNA as Phytophthora endornavirus 2 (PEV2) and the 13.8 kb dsRNA as Phytophthora endornavirus 3 (PEV3). The complete sequences were deposited in the Genbank (NCBI) database with accession numbers LC586217 (PEV2; 14,345 nt) and LC586218 (PEV3, 13,810 nt). The genome organizations of PEV2, PEV3, and PEV1 (Hacker et al., 2005) are illustrated in Figure 2. The PEV2 genome consists of a 379 bp 5′ untranslated region (UTR), a single ORF of 4,640 codons, and a 46 bp 3′ UTR, ending with 8 cytosine residues. The PEV3 genome has a 181 bp 5′ UTR, an ORF with 4,591 codons, and a 37 bp 3′ UTR, which also ends with 8 cytosine residues. These structures are very similar to that of PEV1. PEV1, and PEV3 are each also characterized by the lack of a stop codon in the 5′ UTR (Figures 2B,C).

The polyproteins encoded by the ORFs of PEV2 and PEV3 contain conserved protein domains, which we identified by searching the NCBI Conserved Domain Database (CDD; Marchler-Bauer et al., 2017). These conserved domains in the polyproteins of both viruses include a viral helicase (Superfamily 1; CDD accession pfam01443), a UDP:flavonoid glycosyltransferase (YjiC, YdhE family; CDD accession COG1819), a UDP glycosyltransferase (GT1_Gtf-like; CDD accession cd03784), and an RNA-dependent RNA polymerase 2 (RdRp 2; CDD accession pfam00978). The positions of the helicase (Hel-1), the UDP glycosyltransferase (UGT), and the RdRp in PEV1, PEV2, and PEV3 are shown in Figure 2. The conserved UDP:flavonoid glycosyltransferase domains were located at amino acid position 3,100–3,260 in PEV2 and position 2,991–3,108 in PEV3. The *E*-values indicating similarity to the conserved domains in the database were as follows: Hel-1, 1.97E-05 for PEV2 and 1.24E-08 for PEV3; UGT, 3.91E-04 for PEV2 and 1.73E-04 for PEV3; RdRp, 5.78 E-20 for PEV2 and 2.66E-14 for PEV3; and for the UDP:flavonoid glycosyltransferase, 1.14E-06 for PEV2 and 3.17E-06 for PEV3.

We found low levels of identity (11.2%) and similarity (51.8%) between the complete amino acid sequences of the polyproteins encoded by PEV2 and PEV3. The polyprotein of PEV3 shares 40% amino acid identity with that of PEV1, which infects *Phytophthora* taxon douglasfir (Genbank accession YP_241110.1; Hacker et al., 2005). The identities between the Hel-1, UGT, and RdRp 2 domains of PEV2 and PEV3 are 42, 21, and 53%, respectively. The RdRp and Hel-1 regions of PEV2 and PEV3 show 25–75% identity with the RdRp and Hel-1 domains of other endornaviruses from plants, fungi, and oomycetes (Supplementary Tables 3, 4). On the other hand, we found no similarity between the UGT regions of PEV2 and PEV3 and those of other previously reported endornaviruses. Their UGT regions show 20–40% similarity with the reported UGT regions of bacteria and fungi (data not shown).

### Phylogenetic Analysis of the Putative RdRps Encoded by the PEV2 and PEV3 dsRNAs

**FIGURE 3:**
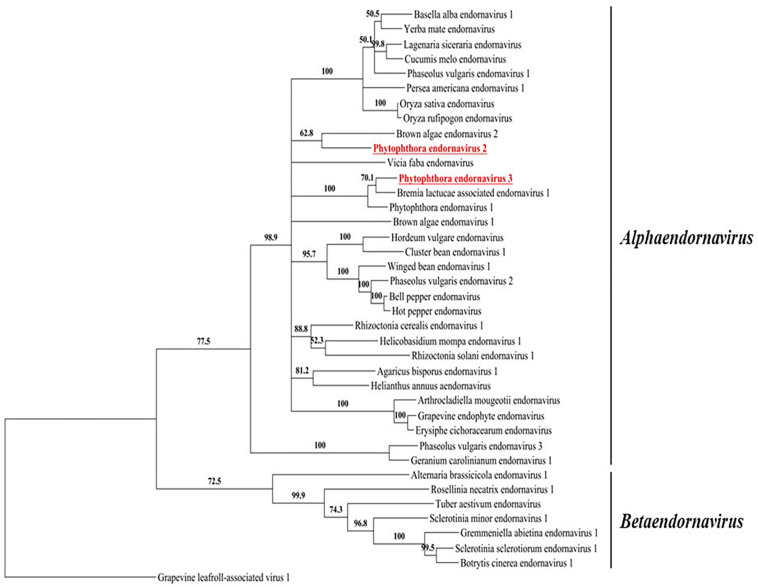
A phylogenic tree based on the putative RdRp regions of PEV2, PEV3, and related endornaviruses. The evolutionary history was inferred by using the Maximum Likelihood method based on the Le_Gascuel_2008 model (Le and Gascuel, 2008). A discrete Gamma distribution was used to model evolutionary rate differences among sites [5 categories (+*G*, parameter = 1.2113)]. The rate variation model allowed for some sites to be evolutionarily invariable ([+*I*], 4.0463% sites). Evolutionary analyses were conducted in MEGA6 (Tamura et al., 2013). Support for nodes was assessed by a reliability percentage after 1,000 bootstrap iterations. Branches with less than 50% bootstrap values were collapsed with TreeGraph 2 ver. 2.14.0-771 beta (Stöver and Müller, 2010). The GenBank accession numbers of the analyzed genes are provided in Supplementary Table 5. Grapevine leafroll-associated virus 1 is an ampelovirus in the family *Closteroviridae* and was used as an outgroup. Undefined virus names are shown in italics.

We constructed a maximum likelihood-based phylogenic tree using the putative RdRp regions of PEV2, PEV3, and 36 related endornaviruses, with Grapevine leafroll-associated virus 1 as the outgroup. The Genbank accession numbers of the viral-encoded sequences used in the analysis are listed in Supplementary Table 5. The tree was constructed using MEGA v. 6.0 (Tamura et al., 2013) and the bootstrap test was performed with 1,000 resamplings (Figure 3). The tree indicated that the endornaviruses could be divided in two clades representing the genera *Alphaendornavirus* and *Betaendornavirus*. PEV2 and PEV3 (along with PEV1) fell within the genus *Alphaendornavirus*. PEV1, PEV2, and PEV3 belong to different virus species. Thus, the phylogenetic analysis supported the classification of PEV2 and PEV3 as new members of the family *Endornaviridae*.

### Transmission of PEV2 and PEV3 via Monozoospores

**FIGURE 4:**
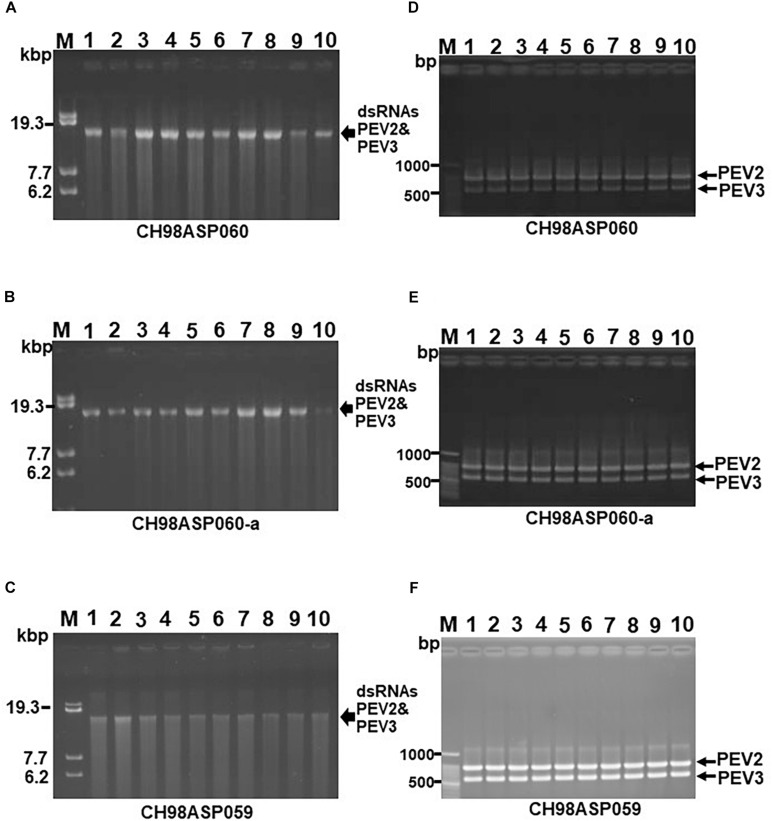
Detection of the PEV2, PEV3 dsRNAs in monozoospore isolates. **(A–C)** Agarose gel electrophoresis of the dsRNA genomes of PEV2 and PEV3 in CH98ASP060 **(A)**, CH98ASP060-a **(B)**, and CH98ASP059 **(C)**. The dsRNAs derived from 0.1 g dry weight of each isolate were electrophoresed in 0.8% agarose gels for 16 h at 20 V and stained with ethidium bromide (0.5 μg/ml). Arrows indicate the positions of the PEV2 and PEV3 dsRNAs. **(D–F)** RT-PCR detection of the PEV2 and PEV3 dsRNA genomes in CH98ASP060 **(D)**, CH98APS060-a **(E)**, and CH98ASP059 **(F)**. RT-PCR was performed using specific primers to amplify the PEV2 and PEV3 dsRNA genomes. The amplified DNA fragments were subjected to electrophoresis in 1% agarose gels for 0.5 h at 100 V. Lane M, 100 bp DNA ladder. Lanes 1–10, individual monozoospore isolates designated numbers 1–10.

To investigate the transmission efficiency of PEV2 and PEV3 via monozoospores, zoosporangia were induced to form on the edges of inoculated filter paper pieces that were incubated on PDA medium containing a low concentration of β-sitosterol (25 μg/ml). Release of the monozoospores was induced by incubating the paper pieces at 18°C in rainwater with gentle shaking. The zoospore suspensions were spread on fresh PDA medium, and after germination, individual germinating hyphae were transferred to flasks, and the mycelia were grown for dsRNA extraction. We cultivated mycelia from 102, 68, and 20 monozoospores derived from isolates CH98ASP060, CH98ASP060-a, and CH98ASP059, respectively. We then used both gel electrophoresis and RT-PCR with specific primers to tested for the presence of the PEV2 and PEV3 dsRNAs in each mycelial clone. All of the mycelial clones contained both the PEV2 and PEV3 dsRNAs. The results indicated that the two dsRNAs were stably transmissible via monozoospores at nearly 100% efficiency, and that no virus-free clones were formed during either zoosporangium formation or zoospore germination. Representative data are shown in Figure 4.

### Effects of the Presence of PEV2 and PEV3 in the Host Vegetative Growth and Developmental Stages

**FIGURE 5:**
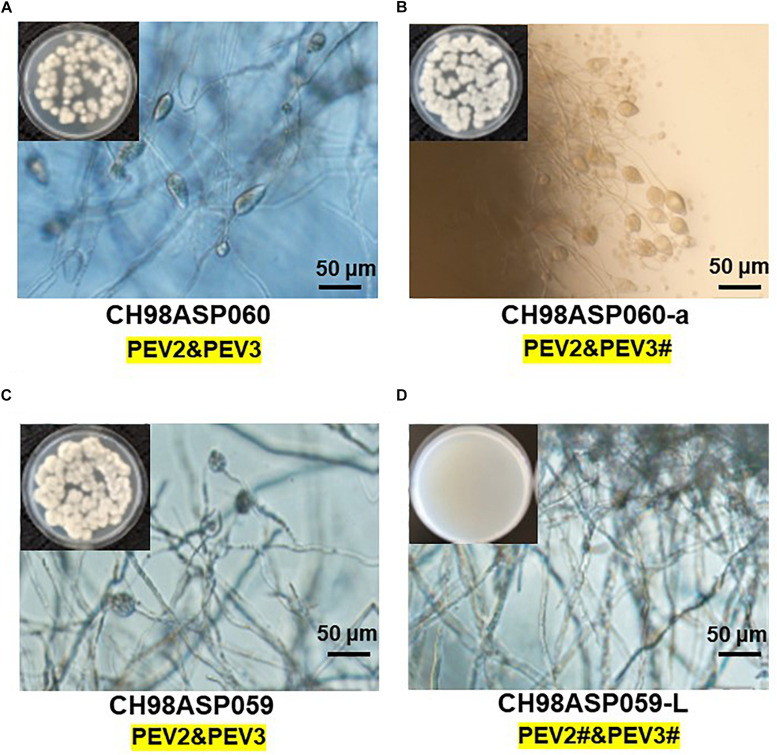
The presence of PEV2 and PEV3 stimulates sporangium production in *Phytophthora* sp. Images show zoosporangium formation by the high-titer isolates CH98ASP060 **(A)**, CH98ASP060-a **(B)**, and CH98ASP059 **(C)** and the low-titer isolate CH98ASP059-L **(D)**. Inserts show hyphal growth on PDA after incubation at 25°C for 7 days. Bars show 50 μm. The # in the dsRNA label under the isolate name is not visible by EtBr staining (0.5 mg/ml) but is detected by RT-PCR. PEV2; 14.3 kb, PEV3; 13.8 kb. This is used in the same mean for the subsequent Figures 6–9.

Five of the original *Phytophthora* sp. isolates from asparagus, along with the sub-isolates CH98ASP060-a and CH98ASP059-L, were grown on PDA medium to compare their hyphal growth, zoosporangium formation, and cystospore germination rates (Table 2). Among the isolates examined, CH98ASP059-L exhibited extremely low titers of PEV2 and PEV3 (Figure 1A) and produced larger mycelial colonies when compared with CH98ASP060, CH98ASP060-a, CH98ASP059, Ku-1, Ak-6-1, and Fk-3 (Table 2 and Figure 1B). Aside from the hyphal growth rates, the most profound differences between these seven isolates were observed in the zoosporangium numbers. Isolate CH98ASP059, which exhibited high titers of PEV2 and PEV3, produced abundant zoosporangia whereas the low-titer isolate CH98ASP059-L formed no zoosporangia (Table 2 and Figure 5). The cystospore germination rates were not significantly different among the six isolates that produced zoosporangia. These results imply that accumulation of high PEV2 and PEV3 levels contributes to reduced hyphal growth and elevated zoosporangium formation in *Phytophthora* sp. infecting asparagus.

**TABLE 2:** Biological effects of PEV2 and PEV3 on vegetative and developmental growths of *Phytophthora* sp.

**isolate (dsRNA)**	**Hyphal growth (mm)^a^**	**Zoosporangium formation^b^**	**Cystospore suspension**	**hyphal germination from cystospore (%)^c^**
CH98ASP060 (PEV2&PEV3)	14.8	21.7 ± 7.6	1 × 10^4^	75.0
CH98ASP060-a (PEV2&*PEV*3#)^d^	21.5	193.0 ± 32.5**	2 × 10^4^	76.0
CH98ASP059 (PEV2&PEV3)	21.9	193.0 ± 37.6**	1 × 10^4^	100.0
CH98ASP059-L (PEV2#&*PEV*3#)^d^	33.0	0.0	–	–
Ku-1 (14 kb-dsRNAs)	20.3	214.0 ± 103.0**	2 × 10^4^	93.0
Ak-6-1 (PEV2&PEV3)	22.2	23.7 ± 7.5	1 × 10^4^	80.0
Fk-3 (PEV2&PEV3)	19.0	134.0 ± 28.1*	2 × 10^4^	83.0

^*a*^The diameter was measured after 7 days of culture on PDA medium (n = 3). ^*b*^The number of zoosporangia occurring around the four edges of a filter paper (1 cm × 1 cm) after 40 h of incubation in rainwater at 18°C. Data are means ± SD from nine filter papers (n = 9). ^*c*^Suspension including 1 × 10^4^ cystospores was spread on PDA medium and grown at 25°C for 5 days. ^*d*^# Invisible by EtBr staining (0.5 mg/ml) but detected by RT-PCR. PEV2; 14.3 kb, PEV3;13.8 kb. * and ** indicate p-values of <0.05 and 0.01, respectively (the Tukey–Kramer range test).

### PEV2 and PEV3 Modulated the Fungicide Sensitivities of the Host Oomycetes

**FIGURE 6:**
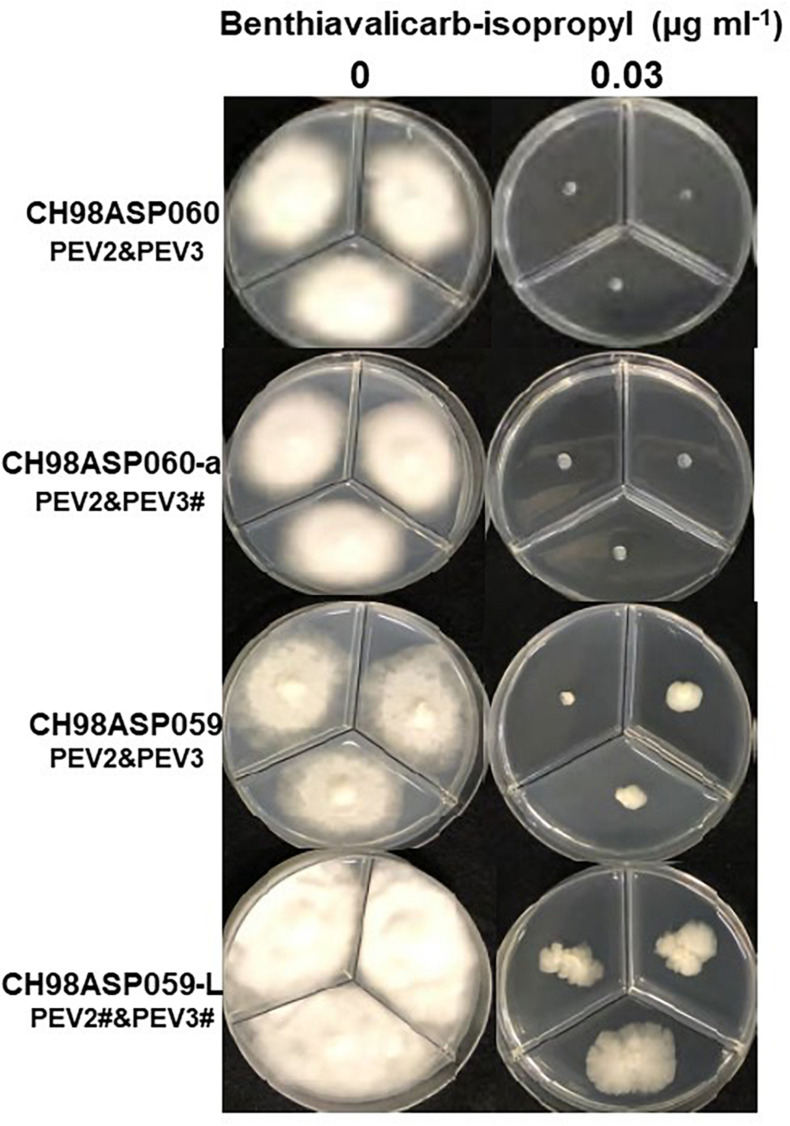
Hyphal growth in on media containing benthiavalicarb-isopropyl (0.03 μg ml^–1^). CH98ASP060, CH98ASP060-a, CH98ASP059, and CH98ASP059-L were grown in the presence **(right)** or absence **(left)** of the fungicide. The minimum inhibitory concentration (MIC) for the high-titer isolates (CH98ASP060, CH98ASP060-a, and CH98ASP059) was used.

**FIGURE 7:**
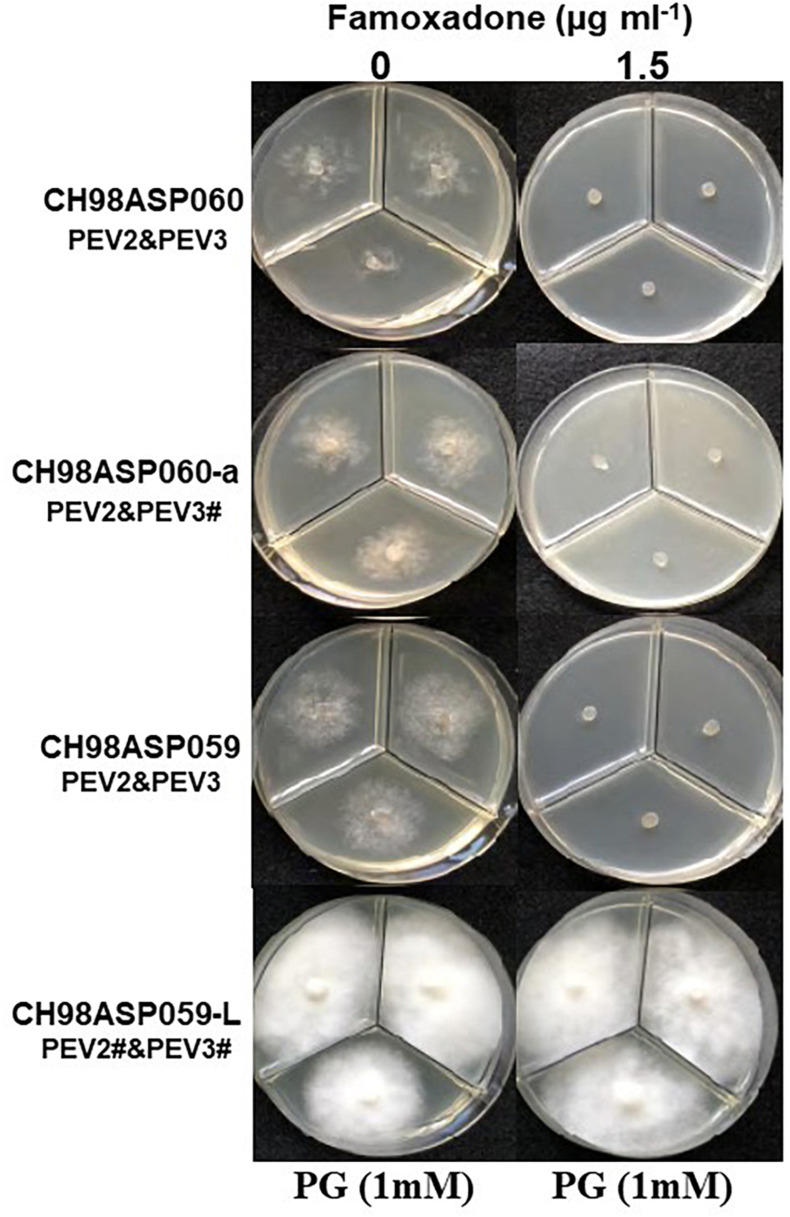
Hyphal growth on media containing famoxadone (1.5 μg ml^–1^). CH98ASP060, CH98ASP060-a, CH98ASP059, and CH98ASP059-L were grown in the presence **(right)** or absence **(left)** of the fungicide. The MIC for the high-titer isolates was used. PG (1 mM) was included in all media.

**FIGURE 8:**
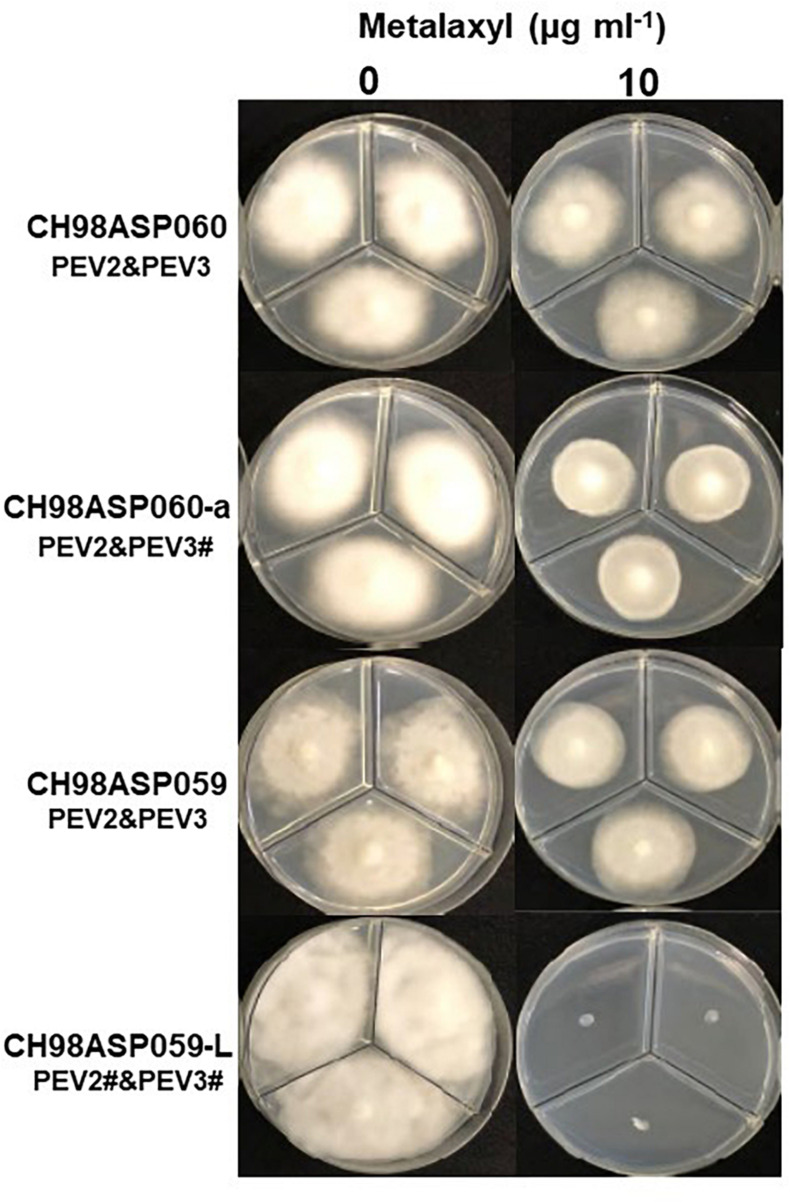
Hyphal growth on media containing metalaxyl (10 μg ml^–1^). CH98ASP060, CH98ASP060-a, CH98ASP059, and CH98ASP059-L were grown in the presence **(right)** or absence **(left)** of the fungicide. The MIC for the low-titer isolate (CH98ASP059-L) was used.

**FIGURE 9:**
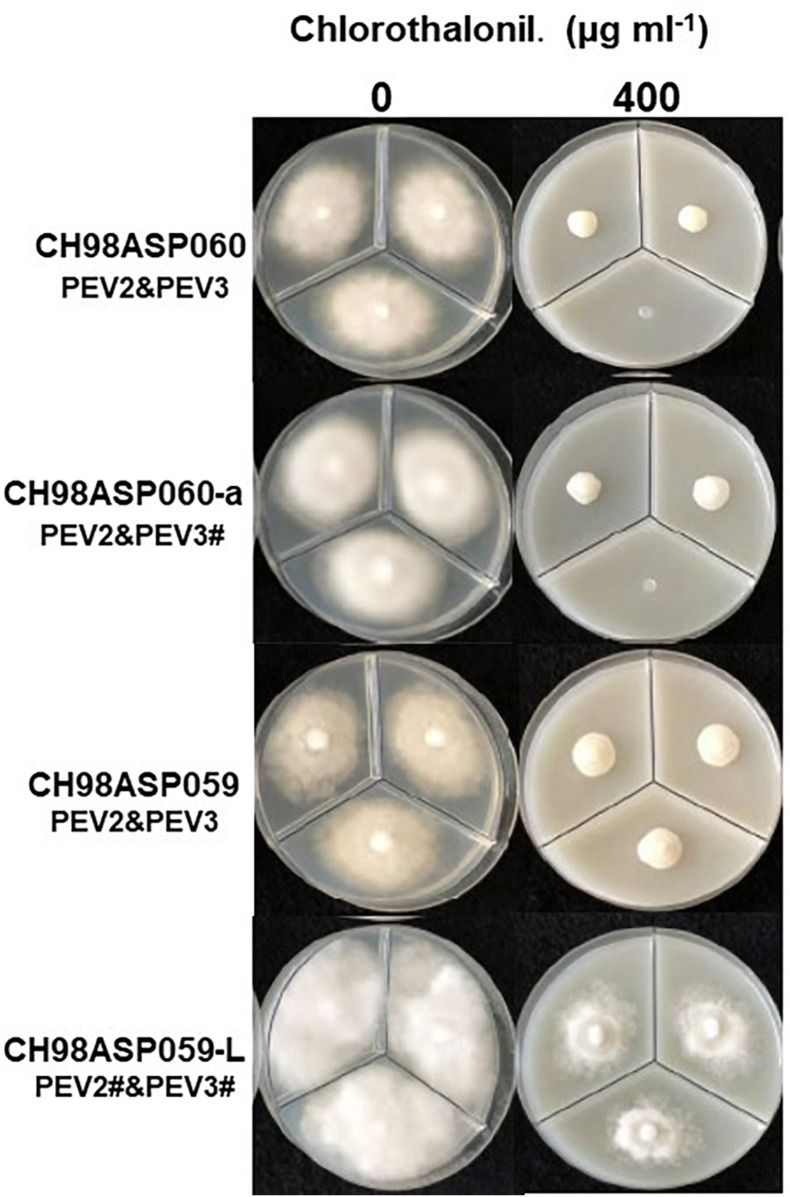
Hyphal growth on media containing chlorothalonil (400 μg ml^–1^). CH98ASP060, CH98ASP060-a, CH98ASP059, and CH98ASP059-L were grown in the presence **(right)** or absence **(left)** of the fungicide. All four isolates had MIC values >400 μg ml^–1^.

We investigated the potential effects of PEV2 and PEV3 infection on the fungicide sensitivities of the asparagus Phytophthora rot pathogens. We used the following four commercially available fungicides: benthiavalicarb-isopropyl (an inhibitor of cellulose synthase), famoxadone (a quinone outside inhibitor that inhibits cytochrome bc1 by binding the quinone binding site), metalaxyl (an inhibitor of RNA polymerase I), and chlorothalonil (an inhibitor of multi-site contact activity). We used serial dilutions of the fungicides in PDA plates and determined the levels of growth inhibition of isolates CH98ASP060, CH98ASP060-a, CH98ASP059, and CH98ASP059-L when compared with their growth on plates containing no fungicide (Tables 3–6). The data were used to determine the minimum inhibitory concentration (MIC) values for each isolate with each fungicide (Supplementary Table 6). Figures 6–9 show hyphal colonies from each isolate, grown on plates containing each fungicide at the MIC for the high-titer isolates, CH98ASP060, CH98ASP060-a, and CH98ASP059.

**TABLE 3:** Sensitivity of *Phytophthora* sp. isolates to benthiavalicarb-isopropyl.

** Isolate**	**Endornaviruses**	**Mycelial growth inhibition (%) at**					
		
		**Benthiavalicarb-isopropyl (μg ml^–1^)**					
		
		**0.003**	**0.03**	**0.3**	**3**	**30^a^**	**150^b^**
CH98APS060	PEV2&PEV3	34.8	100.0	100.0	100.0	100.0	100.0
CH98APS060-a	PEV2&*PEV*3#^c^	8.8	100.0	100.0	100.0	100.0	100.0
CH98ASP059	PEV2&PEV3	0.0	95.4	100.0	100.0	100.0	100.0
CH98ASP059-L	PEV2#&*PEV*3#^c^	3.8	81.8*	100.0	100.0	100.0	100.0

^*a*^The recommended concentration for commercial use. ^*b*^Five times the recommended concentration for commercial use. ^*c*^# Invisible by EtBr staining (0.5 mg/ml) but detected by RT-PCR. PEV2; 14.3 kb, PEV3;13.8 kb * indicate groups that differ significantly (p < 0.05) according to the Tukey–Kramer range test.

**TABLE 4:** Sensitivity of *Phytophthora* sp. isolates to famoxadone.

** Isolate**	** Endornaviruses**	**Mycelial growth inhibition (%) at**						
		
		**Famoxadone (μg ml^–1^) + 1 mM PG**						
		
		**0.0015**	**0.015**	**0.15**	**1.5**	**15**	**150^a^**	**300^b^**
CH98APS060	PEV2&PEV3	23.5	18.5	18.5	100.0	100.0	100.0	100.0
CH98APS060-a	PEV2&*PEV*3#^c^	0.0	15.8	12.6	100.0	100.0	100.0	100.0
CH98ASP059	PEV2&PEV3	0.0	0.0	2.1	100.0	100.0	100.0	100.0
CH98ASP059-L	PEV2#&*PEV*3#^c^	0.0	0.0	0.0	14.1*	21.5	32.3*	27.9*

^*a*^The recommended concentration for commercial use.^*b*^Two times the recommended concentration for commercial use. ^*c*^# Invisible by EtBr staining (0.5 mg/ml) but detected by RT-PCR. PEV2; 14.3 kb, PEV3;13.8 kb. * indicate groups that differ significantly (p < 0.05) according to the Tukey–Kramer range test.

**TABLE 5:** Sensitivity of *Phytophthora* sp. isolates to metalaxyl.

** Isolate**	** Endornaviruses**	**Mycelial growth inhibition (%) at**						
		
		**Metalaxyl (μg ml^–1^)**						
		
		**0.001**	**0.01**	**0.1**	**1**	**10**	**100^a^**	**500^b^**
CH98APS060	PEV2&PEV3	0.0	9.4	6.6	17.9	23.6*	26.4	92.1
CH98APS060-a	PEV2&*PEV*3#	0.0	8.0	8.0	11.5	19.5*	20.7	97.7*
CH98ASP059	PEV2&PEV3	0.0	21.0	24.0	43.0	28.9*	68.0*	93.2
CH98ASP059-L	PEV2#&*PEV*3#	0.0	11.1	62.1	92.2	100.0*	100.0*	100.0*

^*a*^The recommended concentration for commercial use. ^*b*^Five times the recommended concentration for commercial use. * indicate groups that differ significantly (p < 0.05) according to the Tukey–Kramer range test.

**TABLE 6:** Sensitivity of *Phytophthora* sp. isolates to chlorothalonil.

** Isolate**	**Endornaviruses**	**Mycelial growth inhibition (%) at**						
		
		**Chlorothalonil (μg ml^–1^)**						
		
		**0.004**	**0.04**	**0.4**	**4**	**40**	**400^a^**	**800^b^**
CH98APS060	PEV2&PEV3	0.0	1.2	27.9	48.2	82.6	90.7	92.7
CH98APS060-a	PEV2&*PEV*3#	0.0	0.0	25.2	74.4	68.3	90.2	93.5
CH98ASP059	PEV2&PEV3	0.0	0.0	20.5	50.8	73.8	81.1	82.8
CH98ASP059-L	PEV2#&*PEV*3#	2.2	1.4	0.8	10.6	28.6*	47.1*	64.4*

^*a*^The recommended concentration for commercial use. ^*b*^Two times the recommended concentration for commercial use. * indicate groups that differ significantly (p < 0.05) according to the Tukey–Kramer range test.

The high-titer isolates (CH98ASP060, CH98ASP060-a, and CH98ASP059) were highly sensitive to benthiavalicarb-isopropyl and exhibited almost 100% growth inhibition at the concentration of 0.03 μg ml^–1^ (Figure 6 and Table 3). On the other hand, the MIC value for the low-titer isolate (CH98ASP059-L) was 0.3 μg ml^–1^ (Table 3 and Supplementary Table 6). A similar trend was observed with famoxadone: the high-titer isolates had MIC values of 1.5 μg ml^–1^ while the MIC for CH98ASP059-L was more than 150 μg ml^–1^ (Figure 7, Table 4, and Supplementary Table 6). Therefore, the high-titer isolates CH98ASP060, CH98ASP060-a, and CH98ASP059 showed higher susceptibility to both benthiavalicarb-isopropyl and famoxadone, suggesting that the presence of high levels of PEV2 and PEV3 in the host oomycetes increased their sensitivity to these fungicides. The data for chlorothalonil showed a similar, though less dramatic trend. At the recommended concentration for commercial use (400 μg ml^–1^), the high-titer isolates CH98ASP060, CH98ASP060-a, and CH98ASP059 showed growth inhibition rates of 90.7, 90.2, and 81.1%, respectively (Table 6, Figure 9, and Supplementary Table 6). The growth inhibition for CH98ASP059-L was about half of these rates, at 47.1% (Table 6 and Figure 9). Therefore, the presence of high amounts of PEV2 and PEV3 may also increase the sensitivity of these oomycetes to chlorothalonil.

In contrast, the low-titer isolate CH98ASP059-L was more sensitive to metalaxyl than the three high-titer isolates. At the recommended concentration for commercial use (100 μg ml^–1^), the high-titer isolates CH98ASP060, CH98ASP060-a, and CH98ASP059 showed growth inhibition rates of 26.4, 20.7, and 68.0%, respectively (Figure 8 and Table 5). On the other hand, the MIC for CH98ASP059-L was only 10 μg ml^–1^ metalaxyl (Figure 8, Table 5, and Supplementary Table 6). Therefore, the higher amounts of PEV2 and PEV3 in the host oomycetes appear to have reduced their sensitivity to metalaxyl, which is an inhibitor of RNA polymerase I.

### DsRNA Contents of PEV2 and PEV3 in the Host Oomycete Exposed to Metalaxyl

The continuous use of one fungicide or a group of fungicides with similar chemical properties can result in reduced sensitivity in the target organisms. In our fungicide sensitivity tests, the high-titer isolates showed reduced sensitivity to metalaxyl when compared with the low-titer isolate. We conducted an experiment to investigate whether the levels of PEV2 and PEV3 changed in the presence of metalaxyl (Supplementary Figure 2). The three high-titer isolates CH98ASP060, CH98ASP060-a, and CH98ASP059 were sub-cultured five times on PDA plates supplemented with either 0 or 100 μg ml^–1^ metalaxyl. We then grew the mycelia in liquid culture, isolated the dsRNAs, and examined them by gel electrophoresis and RT-PCR. A semiquantitative image system (Ez-Capture MG ATTO, Japan) was used to estimate the amounts of dsRNA in the agarose gels (see the Supplementary Figure 2 legend). The results indicated that under the recommended concentration of 100 μg ml^–1^ metalaxyl, the levels of dsRNA were not significantly different between mycelia sub-cultured in the presence or absence of metalaxyl (Supplementary Figure 2A). The RT-PCR results showed that PEV2 and PEV3 were retained during culture in the presence and absence of metalaxyl (Supplementary Figure 2B).

### Effects of PEV2 and PEV3 on Mycelial Growth in the Presence of *n*-Propyl Gallate

Generally, *n*-propyl gallate (PG) is used in fungicide sensitivity tests with quinone outside inhibitors such as famoxadone, to inhibit the activity of cyanide-insensitive alternative oxidase (AOX) (Hollomon et al., 2005; Ishii et al., 2009). In our sensitivity tests with famoxadone, we included 1 mM PG in all media, including the control medium containing 0 μg ml^–1^ famoxadone. We found that the high-titer isolates appeared to show some growth inhibition in presence of PG when compared with growth in non-supplemented media (compare Figure 7 with Figures, 6, 8, 9). Therefore, we performed sensitivity tests with famoxadone in the absence of PG to determine the effect of famoxadone alone. Interestingly, in these tests the low-titer isolate showed no growth inhibition, even at the maximum concentration of 300 μg ml^–1^ famoxadone. The high-titer isolates showed some growth inhibition, but the inhibition was lower than when PG was included in the media (compare Table 4 with Supplementary Table 7). We then determined the effect of PG alone on hyphal growth. In the presence of 1 mM PG, the high-titer isolates CH98ASP060, CH98ASP060-a, and CH98ASP059 showed growth inhibition rates of 59.5, 77.1, and 57.6%, respectively, compared with their growth on non-supplemented medium. The low-titer isolate showed a lower rate of growth inhibition (25.0%; Supplementary Table 8). Therefore, the presence of PEV2 and PEV3 at relatively high levels appears to confer increased sensitivity to PG.

## Discussion

In this study, we screened *Phytophthora* sp. collected in Japan for infection by mycoviruses, by looking for dsRNA molecules in their mycelia. We found two novel endornaviruses, Phytophthora endornavirus 2 (PEV2, 14,345 nt) and Phytophthora endornavirus 3 (PEV3, 13,810 nt). Phylogenetic analysis of these and other endornaviruses, based on their encoded RdRp sequences, showed that PEV2 and PEV3 are closely related to Phytophthora endornavirus 1 (PEV1) from *Phytophthora* sp. isolates found in Douglas fir (Hacker et al., 2005) (Figure 3). A case in which three different endornaviruses were found in host organisms belonging to the same genus has also been reported in common bean, *P. vulgaris* (Okada et al., 2013, 2018). RT-PCR analysis of the dsRNA from isolate Ku-1, using PEV2- and PEV3-specific primers, resulted in only faint amplification products (Figure 1C). This suggested that the Ku-1 endornaviruses may have sequences that are similar to, but not identical to those of PEV2 and PEV3. This may indicate that the *Phytophthora* endornaviruses have other similar sister viruses. Interestingly, the phylogenetic analysis revealed that PEV2 and PEV3 are evolutionarily close to *Vicia faba* endornavirus (VfEV) (Figure 3, Pfeiffer, 1998). VfEV is the only plant endornavirus that causes significant damage to the host plant (Grill and Garger, 1981). When VfEV was present at a high titer, the anther of the broad bean *V. faba* exhibited an abnormal shape and was male sterile (Turpen et al., 1988).

When we compared hyphal growth rates between the high-titer isolate CH98ASP059 and its low-titer derivative CH98ASP059-L, we found that the high-titer isolate showed a lower growth rate and reduced hyphal density (Figure 1B and Table 2). Similarly, Plasmopara halstedii virus (PhV), which infects downy mildew, inhibited the growth of host hyphae (Grasse et al., 2013). It is possible that the suppression of vegetative hyphal growth by viruses may prevent the spread of oomycete pathogens in the field.

Interestingly, the high-titer isolate CH98ASP059 produced abundant zoosporangia, while the low-titer isolate CH98ASP059-L showed vigorous hyphal growth but produced extraordinarily few zoosporangia. These results suggest that the high accumulation of PEV2 and PEV3 in the host stimulated zoosporangium production (Figure 5 and Table 2). Similarly, Phytophthora infestans RNA virus 2 (PiRV-2) has been reported to stimulate zoosporangium formation (Cai et al., 2019). Zoosporangium formation requires plant sterols such as β-sitosterol. Sterols taken up into oomycete cells are transported from the Golgi apparatus via blastocoel spores, and are thought to play essential roles as materials for constructing zoospore cell membranes (Hendrix, 1970). The high levels of PEV2 and PEV3 in the host oomycetes may contribute to sterol uptake by the host oomycetes.

Phytophthora species are mainly transmitted in the soil, so fungicide management is not easy. Furthermore, the period from infection to spread is very short, and fungicide use has become more frequent over time, leading to reduced efficacy of the fungicides. Therefore, we investigated the sensitivities of our high- and low-titer isolates to four different fungicides. The high titers of PEV2 and PEV3 resulted in high sensitivity to the carboxylic acid amide fungicide, benthiavalicarb-isopropyl (an inhibitor of cellulose synthase). It is known that benthiavalicarb-isopropyl strongly suppresses hyphal growth, direct sporangia germination, cyst germination, and zoosporangium formation (Gisi and Sierotzki, 2015). Assuming that the biosynthesis of the cell wall of the host oomycete is weakened by infection with PEV2 and PEV3, it is possible that the fungicide sensitivity was increased due to increased uptake of the fungicide from the media.

In contrast, high titers of PEV2 and PEV3 resulted in lower sensitivity to metalaxyl (Figure 8, Table 5, and Supplementary Table 6). Metalaxyl is an acylalanine fungicide that functions systemically. It inhibits RNA polymerase I, the uptake of uridine into RNA, and the synthesis of RNA, DNA, and lipids. It affects all developmental stages of the host oomycete including hyphal growth, haustorium formation, and zoosporangium formation (Gisi and Sierotzki, 2015). A possible explanation for the decreased sensitivity to metalaxyl in the high-titer isolates is that the RdRp activities of PEV2 and PEV3 complemented the RNA polymerase I activity of the host during metalaxyl treatment. Suppression of ribosomal RNA synthesis in the host oomycete may make it easier for these endornaviruses to use the RNA substrates. Alternatively, it is also possible that these endornaviruses may autolyze to supply substrates for RNA synthesis by the host oomycete.

It is rare to find such contrasting effects of different fungicides, and it will be interesting to unveil the mechanisms that explain the differences in our results between metalaxyl and the other three fungicides used in this study. To date, few studies have investigated the effects of mycoviruses on host sensitivity to fungicides. It was reported that when Penicillium digitatum polymycovirus 1 (PdPmV1) and Penicillium digitatum Narna-like virus 1 (PdNLV1) co-infected *Penicillium digitatum*, the host showed increased sensitivity to prochloraz (Niu et al., 2018). It may be necessary to consider the presence of viruses in host organisms when examining fungicide sensitivities.

Similar to the case with benthiavalicarb-isopropyl, the high titers of PEV2 and PEV3 resulted in higher sensitivity to famoxadone, an inhibitor of mitochondrial respiration (Figure 7 and Table 4). In this experiment, PG, an inhibitor of cyanide-insensitive alternative oxidase (AOX), was added to the media (Hollomon et al., 2005; Ishii et al., 2009). We found that the high-titer infection of PEV2 and PEV3 conferred increased sensitivity to PG alone (Figure 7 left panels and Supplementary Table 8). AOX is a ubiquinol oxidase that exists on the matrix side of the inner mitochondrial membrane. It is generally present in plants and has also been found broadly in fungi, protozoa, and other lower eukaryotes (Moore et al., 2013). In the future, we would like to investigate the potential mechanisms by which endornaviruses affect the PG sensitivity of AOX in host oomycetes.

In this study, we compared high- and low-titer isolates to evaluate the effects of PEV2 and PEV3 infection on the growth, development, and fungicide sensitivities of the *Phytophthora* sp. isolates from asparagus. Ideally, an isolate with no virus present would have been a better control than the low-titer isolate, however, complete curing of PEV2 and PEV3 appears to be difficult. In addition to the monozoospore isolations described here, we attempted to obtain endornavirus-free clones derived from the low-titer isolate CH98ASP059-L by using the hyphal breakage method (Kim et al., 2012). We assessed 102 single zoospore isolates and 30 colony isolates but found no virus-free clones (data not shown). We also investigated about 30 colony isolates after protoplastization but again, endornaviruses-free hyphae were not obtained (data not shown). The highly efficient vertical transmission of PEV2 and PEV3 via zoospores is reminiscent of the high efficiency of seed transmission of the plant endornaviruses. For example, Oryza sativa endornavirus (OsEV) is mostly localized in the cytoplasm, but seed transmission rates through eggs or pollen were almost 100% (Moriyama et al., 1996). The highly efficient transmission of the endornaviruses during vegetative growth seems to be related to the intracellular localization of the endornaviruses. In interspecific hybrids between OsEV-infected *Oryza sativa* and *Oryza rufipogon*, or in hybrids between the *japonica* and *indica* varieties of *O. sativa*, some F2 individuals were OsEV-free, and this was inherited in a non-Mendelian fashion (Moriyama et al., 1999a,b; Horiuchi et al., 2003). Given these findings with plant endornaviruses, it may be possible to isolate virus-free variants of the *Phytophthora* sp. isolates from asparagus by creating interspecific hybrid cells. This may be achieved by hyphal or protoplast fusion.

## Data Availability Statement

The datasets generated for this study can be found in online repositories. The names of the repository/repositories and accession number(s) can be found in the article/ Supplementary Material.

## Author Contributions

SU collected the materials. KU, KS, and AI performed the experiments with academic and technical assistance from KK, TA, TF, and SU. RO, YT, YK, TO, and TM contributed to the DNA sequencing experiments and phylogenetical analyses. KU and HM analyzed the data and wrote the first draft of the manuscript. All authors critically reviewed the manuscript and approved the final submission.

## Conflict of Interest

The authors declare that the research was conducted in the absence of any commercial or financial relationships that could be construed as a potential conflict of interest.

## References

[B2] Adams M. J. Lefkowitz E. J. King A. M. Q. Harrach B. Harrison R. L. Knowles N. J. (2017). Changes to taxonomy and the international code of virus classification and nomenclature ratified by the international committee on taxonomy of viruses. *Arch. Virol*. 162 2505–2538. 10.1007/s00705-017-3358-5 28434098

[B3] Aoki N. Moriyama H. Kodama M. Arie T. Teraoka T. Fukuhara T. (2009). A novel mycovirus associated with four double-stranded RNAs affects host fungal growth in *Alternaria alternate*. *Virus Res.* 140 179–187. 10.1016/j.virusres.2008.12.003 19118588

[B4] Aragon-Caballero L. M. Hurtado-Gonzales O. P. Flores-Torres J. G. Apaza-Tapia W. Lamour K. H. (2008). First report of *Phytophthora nicotianae* causing asparagus spear and root rot in peru. *Plant Dis*. 92 982.2–982.2. 10.1094/PDIS-92-6-0982B 30769751

[B5] Ark P. A. Barret J. T. (1938). Phytophthora rot of asparagus in California. *Phytopathology* 28 754–756.

[B6] Blair J. E. Coffey M. D. Park S.-Y. Geiser D. M. Kang S. (2008). A multi-locus phylogeny for *Phytophthora* utilizing markers derived from complete genome sequences. *Fungal Genet. Biol*. 45 266–277. 10.1016/j.fgb.2007.10.010 18039586

[B7] Cai G. Krychiw J. F. Myers K. Fry W. E. Hillman B. I. (2013). A new virus from the plant pathogenic oomycete *Phytophthora infestans* with an 8 kb dsRNA genome: the sixth member of a proposed new virusgenus. *Virology* 435 341–349. 10.1016/j.virol.2012.10.012 23146209

[B8] Cai G. Myers K. Fry W. E. Hillman B. I. (2012). A member of the virus family Narnaviridae from the plant pathogenic oomycete *Phytophthora infestans*. *Arch. Virol*. 157 165–169. 10.1007/s00705-011-1126-5 21971871

[B9] Cai G. Myers K. Fry W. E. Hillman B. I. (2019). Phytophthora infestans RNA virus 2, a novel RNA virus from *Phytophthora infestans*, does not belong to any known virus group. *Arch. Virol*. 164 567–572. 10.1007/s00705-018-4050-0 30343382

[B10] Cai G. Myers K. Hillman B. I. Fry W. E. (2009). A novel virus of the late bligt pathogen, *Phytophthora infestans*, with two RNA segments and a supergroup 1 RNA-dependent RNA polymerase. *Virology* 392 52–61. 10.1016/j.virol.2009.06.040 19632701

[B11] Cavalier-Smith T. Chao E. E.-Y. (2006). Phylogeny and megasystematics of phagotrophic heterokonts (kingdom Chromista). *J. Mol. Evol*. 62 388–420. 10.1007/s00239-004-0353-8 16557340

[B12] Cooke D. E. Drenth A. Duncan J. M. Wagels G. Brasier C. M. (2000). A molecular phylogeny of phytophthora and related oomycetes. *Fungal Genet. Biol*. 30 17–32. 10.1006/fgbi.2000.1202 10955905

[B13] Coutts R. H. A. (2005). First report of an endornavirus in the Cucurbitaceae. *Virus Genes*. 31 361–362. 10.1007/s11262-005-3255-y 16175342

[B14] Crous P. W. Summerell B. A. Shivas R. G. Burgess T. I. Decock C. A. Dreyer L. L. (2012). Fungal planet description sheets: 107-127. *Persoonia* 28 138–182. 10.3767/003158512X652633 23105159 PMC3409410

[B15] Falloon P. G. (1982). Baiting, pathogenicity and distribution of *Phytophthora megasperma* var. *sojae* in New Zealand asparagus soils. *N. Z. J. Agric. Res.* 25 425–429. 10.1080/00288233.1982.10417907

[B16] Frohman M. A. Dush M. K. Martin G. R. (1988). Rapid production of full-length cDNAs from rare transcripts: amplification using a single gene-specific oligonucleotide primer. *Proc. Natl. Acad. Sci. U.S.A*. 85 8998–9002. 10.1073/pnas.85.23.8998 2461560 PMC282649

[B17] Fukuhara T. Koga R. Aoki N. Yuki C. Yamamoto N. Oyama N. (2006). The wide distribution of endornaviruses, large double-stranded RNA replicons with plasmid-like properties. *Arch. Virol*. 151 995–1002. 10.1007/s00705-005-0688-5 16341944

[B18] Ghabrial S. A. Castón J. R. Jiang D. Nibert M. L. Suzuki N. (2015). 50-plus years of fungal viruses. *Virology* 356 479–480. 10.1016/j.virol.2015.02.034 25771805

[B19] Ghabrial S. A. Suzuki N. (2009). Viruses of plant pathogenic fungi. *Annu. Rev. Phytopathol*. 47 353–384. 10.1146/annurev-phyto-080508-081932 19400634

[B20] Gisi U. Sierotzki H. (2015). “Oomycete fungicides: phenylamides, quinone outside inhibitors, and carboxylic acid amides,” in *Fungicide Resistance in Plant Pathogens*, eds Ishii H. Hollomon D. (Tokyo: Springer), 145–174. 10.1007/978-4-431-55642-8_10

[B21] Grasse W. Zipper R. Totska M. Spring O. (2013). *Plasmopara halstedii* virus causes hypovirulence in *Plasmopara halstedii*, the downy mildew pathogen of the sunflower. *Fungal Genet. Biol.* 57 42–47. 10.1016/j.fgb.2013.05.009 23747662

[B22] Grill L. K. Garger S. J. (1981). Identification and characterization of double-stranded RNA associated with cytoplasmic male sterility in *Vicia faba*. *Proc. Natl. Acad. Sci. U.S.A*. 78 7043–7046. 10.1073/pnas.78.11.7043 16593124 PMC349190

[B23] Guindon S. Dufayard J.-F. Lefort V. Anisimova M. Hordijk W. Gascuel O. (2010). New algorithms and methods to estimate maximum-likelihood phylogenies: assessing the performance of PhyML 3.0. *Syst. Biol.* 3 307–321. 10.1093/sysbio/syq010 20525638

[B24] Hacker C. V. Brasier C. M. Buck K. W. (2005). A double-stranded RNA from a *Phytophthora* species is related to the plant endornaviruses and contains a putative UDP glycosyltransferase gene. *J. Gen. Virol*. 86 1561–1570. 10.1099/vir.0.80808-0 15831970

[B25] Hendrix J. W. (1970). Sterols in growth and reproduction of fungi. *Annu. Rev. Phytopathol*. 8 111–130. 10.1146/annurev.py.08.090170.000551

[B26] Hollomon D. W. Wood P. M. Reeve C. Miguez M. (2005). “Alternative oxidase and its impact on the activity of Qo and Qi site inhibitors,” in *Modern Fungicides and Antifungal Compounds IV*, eds Dehne H. W. Gisi U. Kuck K. H. Russell P. E. Lyr H. (Alton: BCPC), 31–34.

[B27] Horiuchi H. Moriyama H. Fukuhara T. (2003). Inheritance of *Oryza sativa* endornavirus in F1 and F2 hybrids between japonica and indica rice. *Genes Genet. Syst*. 78 229–234. 10.1266/ggs.78.229 12893964

[B28] Ikeda K. I. Nakamura H. Matsumoto N. (2003). Hypovirulent strain of the violet root rot fungus *Helicobasidium mompa*. *J. Gen. Plant Pathol*. 69 385–390. 10.1007/s10327-003-0076-5 16417937

[B29] Ishii H. Fountaine J. Chung W.-H. Kansako M. Nishimura K. Takahashi K. (2009). Characterisation of QoI-resistant field isolates of *Botrytis cinerea* from citrus and strawberry. *Pest Manag. Sci*. 65 916–922. 10.1002/ps.1773 19444805

[B30] Kim J.-X. Jung J.-E. Park J.-A. Park S.-M. Cha B.-J. Dae-Hyuk Kim D.-H. (2012). Biological function of a novel chrysovirus, CnV1-BS122, in the Korean Cryphonectria nitschkei BS122 strain. *J. Biosci. Bioeng*. 115 1–3. 10.1016/j.jbiosc.2012.08.007 22938824

[B31] Kliejunas J. T. Ko W. H. (1974). Effect of motility of *Phytophthora palmivora* zoospores on disease severity in Papaya seedlings and substrate colonization in soil. *Phytopathology* 64 426–428. 10.1094/phyto-64-426

[B32] Ko W. H. Chan M. J. (1974). Infection and colonization potential of sporangia, zoospores, and chlamydospores of phytophthora palmivora in soil. *Phytopathology* 64 1307–1309. 10.1094/Phyto-64-1307

[B33] Kodama F. Sonoda T. Kawamura T. Okada T. Fujii N. Nara C. (2015). First report of blight disease of asparagus by *Phytophthora* sp. in clade 6 in Japan. *Plant Dis.* 99:1857. 10.1094/PDIS-02-15-0210-PDN

[B34] Komatsu K. Katayama Y. Omatsu T. Mizutani T. Fukuhara T. Kodama M. (2016). Genome sequence of a novel mitovirus identified in the phytopathogenic fungus Alternaria arborescens. *Arch Virol*. 161 2627–2631. 10.1007/s00705-016-2953-1 27368994

[B35] Kozlakidis Z. Brown N. A. Jamal A. Phoon X. Coutts R. H. A. (2010). Incidence of endornaviruses in *Phytophthora* taxon douglasfir and *Phytophthora ramorum*. *Virus Genes* 40 130–134. 10.1007/s11262-009-0421-7 19915969

[B36] Larkin M. A. Blackshields G. Brown N. P. Chenna R. McGettigan P. A. McWilliam H. (2007). Clustal W and Clustal X version 2.0. *Bioinformatics* 23 2947–2948. 10.1093/bioinformatics/btm404 17846036

[B37] Le S. Q. Gascuel O. (2008). An improved general amino acid replacement matrix. *Mol. Biol. Evol.* 25 1307–1320. 10.1093/molbev/msn067 18367465

[B38] Lefebvre A. Scalla R. Pfeiffer P. (1990). The double-stranded RNA associated with the ‘447’ cytoplasmic male sterility in *Vicia faba* is packaged together with its replicase in cytoplasmic membranous vesicles. *Plant Mol. Biol*. 14 477–490. 10.1007/BF00027494 2102829

[B39] Mackenzie S. A. Pring D. R. Bassett M. J. (1988). Large doublestranded RNA molecules in *Phaseolus vulgaris* L. are not associated with cytoplasmic male sterility. *Theor. Appl. Genet*. 76 59–63. 10.1007/BF00288832 24231983

[B40] Marchler-Bauer A. Bo Y. Han L. He J. Lanczycki C. J. Lu S. (2017). CDD/SPARCLE: functional classification of proteins via subfamily domain architectures. *Nucleic Acids Res.* 45 D200–D203. 10.1093/nar/gkw1129 27899674 PMC5210587

[B41] Marzano S. L. Nelson B. D. Ajayi-Oyetunde O. Bradley C. A. Hughes T. J. Hartman G. L. (2016). Identification of diverse mycoviruses through metatranscriptomics characterization of the viromes of five major fungal plant pathogens. *J. Virol*. 90 6846–6863. 10.1128/JVI.00357-16 27194764 PMC4944287

[B42] Moore A. L. Shiba T. Young L. Harada S. Kita K. Ito K. (2013). Unraveling the heater: new insights into the structure of the alternative oxidase. *Annu. Rev. Plant Biol*. 64 637–663. 10.1146/annurev-arplant-042811-105432 23638828

[B43] Moriyama H. Horiuchi H. Koga R. Fukuhara T. (1999a). Molecular characterization of two endogenous double-stranded RNAs in rice and their inheritance by interspecific hybrids. *J. Biol. Chem.* 274 6882–6888. 10.1074/jbc.274.11.6882 10066741

[B44] Moriyama H. Horiuchi H. Nitta T. Fukuhara T. (1999b). Unusual inheritance of evolutionarily-related double-stranded RNAs in interspecific hybrid between rice plants *Oryza sativa* and *Oryza rufipogon*. *Plant Mol. Biol*. 39 1127–1136. 10.1023/a:1006118304093 10380800

[B45] Moriyama H. Kanaya K. Wang J. Z. Nitta T. Fukuhara T. (1996). Stringently and developmentally regulated levels of a cytoplasmic double-stranded RNA and its high-efficiency transmission via egg and pollen in rice. *Plant Mol. Biol*. 31 713–719. 10.1007/BF00019459 8806402

[B46] Moriyama H. Nitta T. Fukuhara T. (1995). Double-stranded RNA in rice: a novel RNA replicon in plants. *Mol. Gen. Genet.* 248 364–369. 10.1007/BF02191603 7565598

[B47] Niu Y. Yuan Y. Mao J. Yang Z. Cao Q. Zhang T. (2018). Characterization of two novel mycoviruses from *Penicillium digitatum* and the related fungicide resistance analysis. *Sci. Rep*. 8:5513. 10.1038/s41598-018-23807-3 29615698 PMC5882929

[B48] Nuss D. L. (2005). Hypovirulence: mycoviruses at the fungal-plant interface. *Nat. Rev. Microbiol*. 3 632–642. 10.1038/nrmicro1206 16064055

[B49] Okada R. Alcalá-Briseño I. R. Escalante C. Sabanadzovic S. Rodrigo A. Valverde R. A. (2018). Genomic sequence of a novel endornavirus from *Phaseolus vulgaris* and occurrence in mixed infections with two other endornaviruses. *Virus Res.* 257 63–67. 10.1016/j.virusres.2018.09.005 30218691

[B50] Okada R. Kiyota E. Moriyama H. Fukuhara T. Natsuaki T. (2015). A simple and rapid method to purify viral dsRNA from plant and fungal tissue. *J. Gen. Plant Pathol.* 81 103–107. 10.1007/s10327-014-0575-6

[B51] Okada R. Yong C. K. Valverde R. A. Sabanadzovic S. Aoki N. Hotate S. (2013). Molecular characterization of two evolutionarily distinct endornaviruses co-infecting common bean (*Phaseolus vulgaris*). *J. Gen. Virol.* 94 220–229. 10.1099/vir.0.044487-0 23015743

[B52] Ong J. W. L. Li H. Sivasithamparam K. Dixon K. W. Jones M. G. K. Wylie S. J. (2016). Novel endorna-like viruses, including three with two open reading frames, challenge the membership criteria and taxonomy of the Endornaviridae. *Virology* 499 203–211. 10.1016/j.virol.2016.08.019 27677157

[B53] Osaki H. Nakamura H. Sasaki A. Matsumoto N. Yoshida K. (2006). An endornavirus from a hypovirulent strain of the violet root rot fungus, *Helicobasidium mompa*. *Virus Res*. 118 143–149. 10.1016/j.virusres.2005.12.004 16417937

[B54] Pearson M. N. Beever R. E. Boine B. Arthur K. (2009). Mycoviruses of filamentous fungi and their relevance to plant pathology. *Mol. Plant Pathol*. 10 115–128. 10.1111/j.1364-3703.2008.00503.x 19161358 PMC6640375

[B55] Pfeiffer P. (1998). Nucleotide sequence, genetic organization and expression strategy of the double-stranded RNA associated with the ‘447’cytoplasmic male sterility in *Vicia faba*. *J. Gen. Virol*. 79 2349–2358. 10.1099/0022-1317-79-10-2349 9780039

[B56] Pfeiffer P. Jung J. L. Heitzler J. Keith G. (1993). Unusual structure of the double-stranded RNA associated with the ‘447’cytoplasmic male sterility in *Vicia faba*. *J. Gen. Virol*. 74 1167–1173. 10.1099/0022-1317-74-6-1167 7685375

[B57] Saude C. Hurtado-Gonzales O. P. Lamour K. H. Hausbeck M. K. (2008). Occurrence and characterization of a *Phytophthora* sp. pathogenic to asparagus (*Asparagus officinalis*) in Michigan. *Phytopathology* 98 1075–1083. 10.1094/PHYTO-98-10-1075 18943453

[B58] Stöver B. C. Müller K. F. (2010). TreeGraph 2: combining and visualizing evidence from different phylogenetic analyses. *BMC Bioinformatics* 5:7. 10.1186/1471-2105-11-7 20051126 PMC2806359

[B59] Tamura K. Stecher G. Peterson D. Filipski A. Kumar S. (2013). MEGA6: molecular evolutionary genetics analysis version 6.0. *Mol. Biol. Evol*. 30 2725–2729. 10.1093/molbev/mst197 24132122 PMC3840312

[B60] Thompson J. D. Gibson T. J. Plewniak F. Jeanmougin F. Higgins D. G. (1997). The CLUSTAL_X windows interface: flexible strategies for multiple sequence alignment aided by quality analysis tools. *Nucleic Acids Res*. 25 4876–4882. 10.1093/nar/25.24.4876 9396791 PMC147148

[B61] Tokunaga T. Bartnicki-Garcia S. (1971). Cyst wall formation and endogenous carbohy drate utilization during synchronous encystment of *Phytophthora palmivora* zoospores. *Arch. Mikrobiol*. 79 283–292. 10.1007/BF00424905 5126075

[B62] Tuomivirta T. T. Kaitera J. Hantula J. (2009). A novel putative virus of *Gremmeniella abietina* type B (Ascomycota: helotiaceae) has a composite genome with endornavirus affinities. *J. Gen. Virol*. 90 2299–2305. 10.1099/vir.0.011973-0 19494051

[B63] Turpen T. Garger S. J. Grill L. K. (1988). On the mechanism of cytoplasmic male sterility in the 447 line of Vicia faba. *Plant Mol. Biol*. 10 489–497. 10.1007/BF00033604 24277621

[B64] Valverde R. A. Gutierrez D. L. (2007). Transmission of a dsRNA in bell pepper and evidence that it consists of the genome of an endornavirus. *Virus Genes* 35 399–403. 10.1007/s11262-007-0092-1 17393294

[B65] Valverde R. A. Khalifa M. E. Okada R. Fukuhara T. Sabanadzovic S. (2019). ICTV virus taxonomy profile: *Endornaviridae*. *J. Gen. Virol*. 100 1204–1205. 10.1099/jgv.0.001277 31184570 PMC12643110

[B66] Wakarchuk D. A. Hamilton R. I. (1985). Cellular double-stranded RNA in *Phaseolus vulgaris*. *Plant Mol. Biol*. 5 55–63. 10.1007/BF00017873 24306540

[B67] Wakarchuk D. A. Hamilton R. I. (1990). Partial nucleotide sequence from enigmatic dsRNAs in *Phaseolus vulgaris*. *Plant Mol. Biol*. 14 637–639. 10.1007/BF00027512 2102845

[B68] Webster J. Weber R. W. S. (2007). *Introduction to Fungi.* Cambridge: Cambridge University Press 841.

[B69] Xie J. Jiang D. (2014). New insights into mycoviruses and exploration for the biological control of crop fungal diseases. *Annu. Rev. Phytopathol*. 52 45–68. 10.1146/annurev-phyto-102313-050222 25001452

[B70] Yang D. Wu M. Zhang J. Chen W. Li G. Yang L. (2018). Sclerotinia minor endornavirus 1, a novel pathogenicity debilitation-associated mycovirus with a wide spectrum of horizontal transmissibility. *Viruses* 27:589. 10.3390/v10110589 30373273 PMC6266790

