# Supplementary material for: Two Novel Endornaviruses Co-infecting a Phytophthora Pathogen of Asparagus officinalis Modulate the Developmental Stages and Fungicide Sensitivities of the Host Oomycete
Source: Front Microbiol. 2021 Feb 3;12:633502. doi: 10.3389/fmicb.2021.633502 (PMC7902037; doi:10.3389/fmicb.2021.633502)
Supplement: Supplementary file 1 [file Data_Sheet_1.pdf]

Supplementary Material

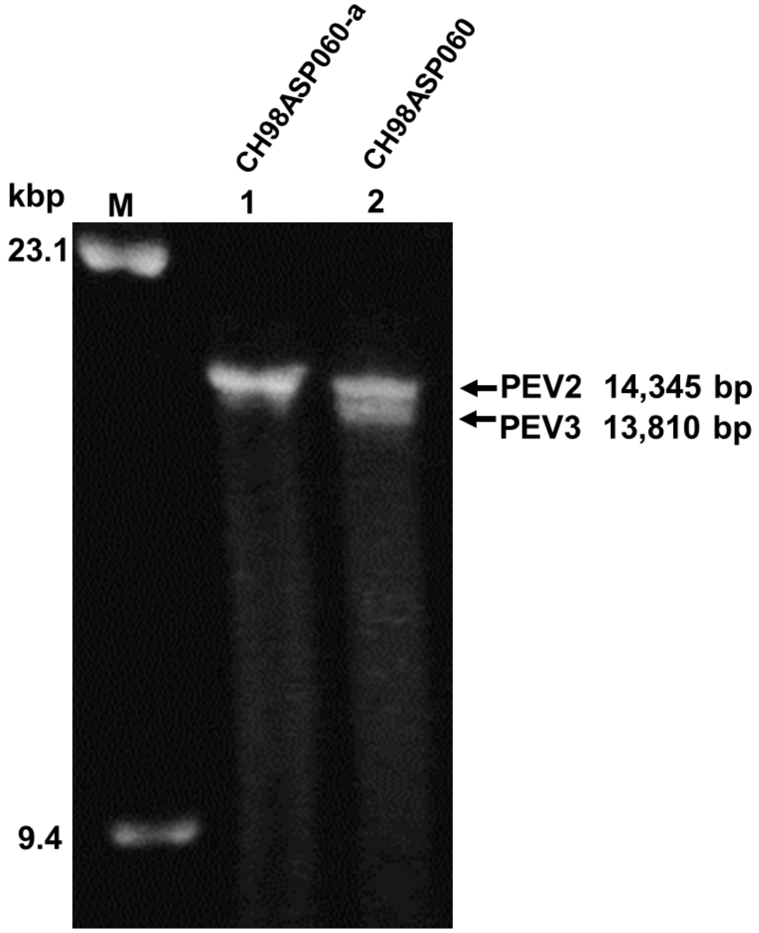

Supplementary Figure 1. Agarose gel electrophoresis of the PEV2 and PEV3 dsRNAs. The electrophoresis conditions were: 0.6 % agarose, 20V, 50h, stained with 0.5 mg/ml ethidium bromide. Lane M, DNA size markers (250 ng of λ DNA digested with Hind III) ; lane 1, CH98ASP060-a/PEV2&PEV3#; lane 2, CH98ASP060/PEV2&PEV3.

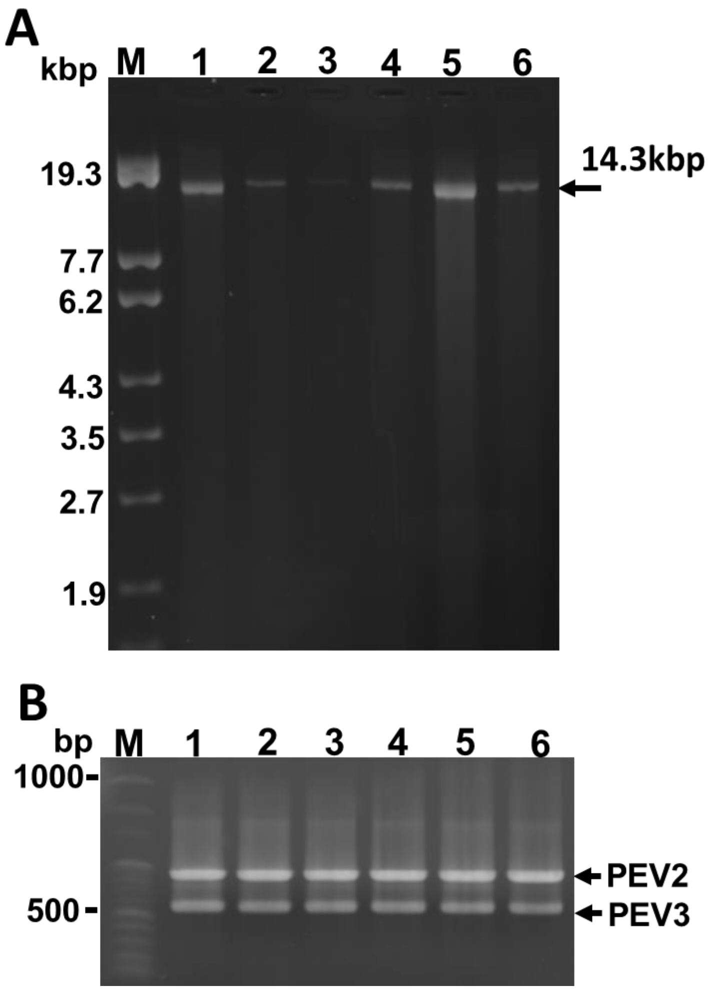

Supplementary Figure 2. Detection of the dsRNA genomes of PEV2 and PEV3 in *Phytophthora* sp. isolates grown on PDA or in PD broth containing 100 µg ml-1 metalaxyl. (A) Agarose gel electrophoresis of dsRNAs purified from the three PEV2 and PEV3 high-titer isolates. The intensities of the bands were semi-quantified by densitometry using a CS-Analyzerver.3.0 (ATTO). Lane 1, 51.2 ng; lane 2, 15.7 ng; lane 3, 5.3 ng; lane 4, 30.0 ng; lane 5, 48.6 ng; lane 6, 35.8 ng. (B) RT-PCR amplification using PEV2 and PEV3-specific primers (Table S2). Lane designations: 1–3, After subculturing 5 times on PDA medium without metalaxyl, the isolates were grown in PD broth under static culture conditions. 4–6, After subculturing 5 times on PDA medium containing 100 mg ml^{-1} metalaxyl, the isolates were grown in PD broth under static culture conditions. M, DNA size markers (300 ng of λ DNA digested with EcoT14I); lanes 1 and 4, CH98ASP060-a/PEV2&PEV3#; lanes 2 and 5, CH98ASP060/PEV2&PEV3; lanes 3 and 6, CH98ASP059/PEV2&PEV3.

Supplementary Table S1. Occurrence of dsRNAs in 68 *Phytophthora* isolates and 2 Pythium isolates.

Species, host	Isolate	Location	dsRNA	Species, host	Isolate	Location	dsRNA
Phytophthora sp.				*P. cactorum*			
Asparagus	CH98ASP060	Toyama	+	Loquat	CH98LOQ1 MAFF242859	Chiba	−
Asparagus	CH98ASP051	Toyama	+	*P. citrophthora*			
Asparagus	CH98ASP059	Toyama	+	Kiwifruit	CH90-15 MAFF245812	Chiba	−
Asparagus	CH98ASP066	Toyama	+	Kiwifruit	CH90-16	Chiba	−
Asparagus	Ku-1	Hokkaido	+	Kiwifruit	CH90-19 MAFF242874	Chiba	−
Asparagus	Ak-6-1	Akita	+	Citrus unshiu	CH98U7B	Chiba	−
Asparagus	Fk-3	Fukushima	+	Citrus unshiu	CH98U8B	Chiba	−
P. nicotianae				*P. cryptogea*			
Carrot	CH08DC9	Okinawa	−	Carnation	CH96CAR51	Chiba	−
Roselle	CH08HS1	Okinawa	−	Carnation	CH96CAR4	Chiba	−
Roselle	CH08HS3	Okinawa	−	Carnation	CH96CAR8	Chiba	−
Roselle	CH08HS7	Okinawa	−	Gerbera	CH95PHG4	Chiba	−
Roselle	CH08HS9	Okinawa	−	Gerbera	CH97PHG9	Chiba	−
Roselle	CH08HS10	Okinawa	−	Eustoma	CH95PHE26	Chiba	−
Kidny bean	CH1999PV14	Chiba	−	Eustoma	CH95PHE27	Ibaraki	−
Lavender	CH99LAV1-1	Chiba	−	Eustoma	CH95PHE10 MAFF245820	Chiba	−
Lavender	CH99LAV1-2	Chiba	−	Eustoma	CH95PHE16 MAFF242880	Ibaraki	−
Strelitzia	CH07STR23	Chiba	−	*P. palmivora*			
Strelitzia	CH07STR25	Chiba	−	Oncidium	CH88-1 MAFF242892	Chiba	−
Strelitzia	CH07STR32	Chiba	−	Cymbidium	CH05PAL051	Chiba	−
Strelitzia	CH07STR34	Chiba	−	Cymbidium	CH06PAL061	Chiba	−
Strelitzia	CH07STR34-2	Chiba	−	Citrus	CH99KP1	Chiba	−
Strelitzia	CH07STR34-3	Chiba	−	Strelitzia	CH07STR12	Chiba	−
Strelitzia	CH07STR42	Chiba	−	Strelitzia	CH07STR14	Chiba	−
Strelitzia	CH07STR43	Chiba	−	*P. citricola*			
Strelitzia	CH07STR42 (4-4)	Chiba	−	Citrus unshiu	CH98U121C	Chiba	−
Strelitzia	CH07STR42 (4-5)	Chiba	−	Citrus unshiu	CH98U122C	Chiba	−
Strelitzia	CH07STR72	Chiba	−	Citrus unshiu	CH98U132C	Chiba	−
Strelitzia	CH07STR81	Chiba	−	*P. cinnamomi*			
Strelitzia	CH07STR101	Chiba	−	Eustoma	CH95PHE1	Ibaraki	−
Citrus junos	CH98Y1A-W-2 MAFF245844	Chiba	−	Eustoma	CH95PHE1-1	Ibaraki	−
Citrus junos	CH98Y1A-W-3 MAFF245844	Chiba	−	Eustoma	CH95PHE4	Ibaraki	−
Citrus junos	CH98Y2A	Chiba	−	Eustoma	CH95PHE20	Ibaraki	−

Citrus junos	CH98Y2A-1	Chiba	–	*Pythium spp.*			
Bougainvillea	CH89-50	Chiba	–	Nemophila	CH98PYNE1	Chiba	–
Dianthus	CH87KY22	Chiba	–	Ranunculus	CH08RAPY9	Chiba	–
Dianthus	CH99KY11	Chiba	–				
Easter lily	CH91PKL3	Chiba	–				
Parsley	CH85PHP105	Chiba	–				
Parsley	CH85PHP61	Chiba	–				
Carnation	CH01DH1	Chiba	–				

+, infection; -, negative.

Supplementary Table S2. List of primers used in the study.

primer name	sequence (5 '-3 ')	position
PEV2-1F	AACCACACTTGCGAGCCTATGAAC	PEV2 (2369-2392)
PEV2-1R	GTCGCCCAGACTAGTTTCAATTGC	PEV2 (3305-3328)
PEV2-5' RACE	GGTCAAGACAGGCGGTAATCTCG	PEV2 (144-166)
PEV2-3' RACE	GTGATGTTCACCAAGCATGGGACGTTCTG	PEV2 (13904-13932)
PEV3-1F	GTTCCAATACCGTCCAACGTGTC	PEV3 (961-983)
PEV3-1R	ACTTCACCAGGGACCTTCATGG	PEV3 (1559-1580)
PEV3-5' RACE	CTTGTACCTATTTGTAAGTCTTTAGGTGATGG	PEV3 (214-245)
PEV3-3' RACE	CACGGCACTTTCTGTAGTATGGTGGCTTAC	PEV3 (13396-13425)
oligo-dT	CGA TGG TAC CTG CAG GCG CGC CTT TTT TTT TTT TTT TTT	for 3' RACE
oligo-dT AP	CGA TGG TAC CTG CAG GCG CGC C	for 3' RACE

Supplementary Table S3. Amino acid identities between the RdRp regions of PEV2 and PEV3 and those of other endornaviruses.

virus name	RdRp		Sequence ID
	PEV2	PEV3	
Lagenaria siceraria endornavirus	-	43%	YP_009010973.1
Cucumis melo endornavirus	43%	45%	YP_009222598.1
Yerba mate endornavirus	45%	46%	YP_009046830.1
Vicia faba endornavirus	49%	44%	YP_438201.1
Phytophthora endornavirus 1	53%	72%	YP_241110.1
Hordeum vulgare endornavirus	43%	44%	YP_009212849.1
Winged bean endornavirus 1	41%	44%	YP_009305414.1
Phaseolus vulgaris endornavirus 2	42%	44%	BAM68540.1
Hot pepper endornavirus	43%	43%	YP_009165596.1
Rhizoctonia cerealis endornavirus 1	48%	46%	YP_008719905.1
Rhizoctonia solani endornavirus 1	43%	-	YP_009552276.1

Arthrocladiella mougeotii endornavirus	-	44%	AZO92732.1
Helianthus annuus endornavirus	47%	43%	YP_009553502.1
Brown algae endornavirus 1	47%	49%	BBZ90073.1
Brown algae endornavirus 2	49%	-	BBZ90074.1
Bremia lactucae associated endornavirus 1	52%	75%	QIP68005.1

Supplementary Table S4. Amino acid identities between the viral helicase (Hel-1) motifs of PEV2 and PEV3 and those of other endornaviruses.

virus name	Hel-1		Sequence ID
	PEV2	PEV3	
Lagenaria siceraria endornavirus	31%	30%	YP_009010973.1
Cucumis melo endornavirus	33%	-	YP_009222598.1
Yerba mate endornavirus	31%	-	YP_009046830.1
Persea americana endornavirus 1	33%	-	YP_005086952.1
Oryza sativa endornavirus	32%	30%	YP_438200.1
Oryza rufipogon endornavirus	-	31%	YP_438202.1
Vicia faba endornavirus	33%	33%	YP_438201.1
Phytophthora endornavirus 1	39%	58%	YP_241110.1
Hordeum vulgare endornavirus	27%	-	YP_009212849.1
Winged bean endornavirus 1	28%	28%	YP_009305414.1
Phaseolus vulgaris endornavirus 2	-	34%	BAM68540.1
Hot pepper endornavirus	-	34%	YP_009165596.1
Rhizoctonia cerealis endornavirus 1	30%	30%	YP_008719905.1
Agaricus bisporus endornavirus 1	-	32%	AQM32768.1
Grapevine endophyte endornavirus	25%	29%	YP_007003829.1
Erysiphe cichoracearum endornavirus	27%	28%	YP_009225663.1
Rhizoctonia solani endornavirus 1	25%	29%	YP_009552276.1
Arthrocladiella mougeotii endornavirus	-	30%	AZO92732.1
Helianthus annuus endornavirus	-	31%	YP_009553502.1
Brown algae endornavirus 1	29%	31%	BBZ90073.1
Brown algae endornavirus 2	32%	33%	BBZ90074.1
Geranium carolinianum endornavirus 1	-	27%	QBB21108.1
Bremia lactucae associated endornavirus 1	39%	63%	QIP68005.1

Supplementary Table S5. GenBank accession numbers of the virus-encoded amino acid sequences used in the phylogenetic analysis**.**

Virus name	Accession	Genus	Family
Lagenaria siceraria endornavirus	YP_009010973.1	*Alphaendornavirus*	*Endornaviridae*
Cucumis melo endornavirus	YP_009222598.1	*Alphaendornavirus*	*Endornaviridae*

Phaseolus vulgaris endornavirus 1	YP_009011062.1	*Alphaendornavirus*	*Endornaviridae*
Basella alba endornavirus 1	AB844264.1	*Alphaendornavirus*	*Endornaviridae*
Yerba mate endornavirus	YP_009046830.1	*Alphaendornavirus*	*Endornaviridae*
Persea americana endornavirus 1	YP_005086952.1	*Alphaendornavirus*	*Endornaviridae*
Oryza sativa endornavirus	YP_438200.1	*Alphaendornavirus*	*Endornaviridae*
Oryza rufipogon endornavirus	YP_438202.1	*Alphaendornavirus*	*Endornaviridae*
Vicia faba endornavirus	YP_438201.1	*Alphaendornavirus*	*Endornaviridae*
Phytophthora endornavirus 1	YP_241110.1	*Alphaendornavirus*	*Endornaviridae*
Hordeum vulgare endornavirus	YP_009212849.1	*Alphaendornavirus*	*Endornaviridae*
Winged bean endornavirus 1	YP_009305414.1	*Alphaendornavirus*	*Endornaviridae*
Phaseolus vulgaris endornavirus 2	BAM68540.1	*Alphaendornavirus*	*Endornaviridae*
Bell pepper endornavirus	YP_004765011.1	*Alphaendornavirus*	*Endornaviridae*
Hot pepper endornavirus	YP_009165596.1	*Alphaendornavirus*	*Endornaviridae*
Helicobasidium mompa endornavirus 1	YP_003280846.1	*Alphaendornavirus*	*Endornaviridae*
Rhizoctonia cerealis endornavirus 1	YP_008719905.1	*Alphaendornavirus*	*Endornaviridae*
Agaricus bisporus endornavirus 1	AQM32768.1	*Alphaendornavirus*	*Endornaviridae*
Grapevine endophyte endornavirus	YP_007003829.1	*Alphaendornavirus*	*Endornaviridae*
Erysiphe cichoracearum endornavirus	YP_009225663.1	NA	NA
Cluster bean endornavirus 1	AYA60157.1	NA	NA
Phaseolus vulgaris endornavirus 3	AXB99512.1	NA	NA
Rhizoctonia solani endornavirus 1	YP_009552276.1	NA	NA
Arthrocladiella mougeotii endornavirus	AZO92732.1	NA	NA
Helianthus annuus endornavirus	YP_009553502.1	NA	NA
Brown algae endornavirus 1	BBZ90073.1	NA	NA
Brown algae endornavirus 2	BBZ90074.1	NA	NA
Geranium carolinianum endornavirus 1	QBB21108.1	NA	NA
Phytophthora endornavirus 2	LC586217	NA	NA
Phytophthora endornavirus 3	LC586218	NA	NA
Bremia lactucae associated endornavirus 1	QIP68005.1	NA	NA
Alternaria brassicicola endornavirus 1	YP_009115493.1	*Betaendornavirus*	*Endornaviridae*
Tuber aestivum endornavirus	YP_004123950.1	*Betaendornavirus*	*Endornaviridae*
Gremmeniella abietina endornavirus 1	YP_529670.1	*Betaendornavirus*	*Endornaviridae*
Sclerotinia sclerotiorum endornavirus 1	YP_009022070.1	*Betaendornavirus*	*Endornaviridae*
Botrytis cinerea endornavirus 1	YP_009315910.1	*Betaendornavirus*	*Endornaviridae*
Rosellinia necatrix endornavirus 1	YP_009276355.1	NA	NA
Sclerotinia minor endornavirus 1	YP_009552723.1	NA	NA
Grapevine leafroll-associated virus 1	YP_004940642	*Ampelovirus*	*Closteroviridae*

NA= Not available.

Supplementary Table S6. Sensitivities of the *Phytophthora* sp. isolates to four fungicides, based on the minimal inhibitory concentrations (MICs).

Isolate	endornaviruses	MIC (μg ml $^{-1}$)			
		benthiavalicarb-isopropyl	famoxadone + 1mM PG	metalaxyl	chlorothalonil
CH98APS060	PEV2&PEV3	0.03	1.5	>100[a]	>400
CH98APS060-a	PEV2&PEV3#	0.03	1.5	>100	>400
CH98ASP059	PEV2&PEV3	0.03	1.5	>100	>400
CH98ASP059-L	PEV2#&PEV3#	0.3	>150	10.0	>400

[a]The MIC value is shown with the recommended concentration as the upper limit.

Supplementary Table S7. Sensitivities of the *Phytophthora* sp. isolates to famoxadone on PDA medium without the addition of n-propyl gallate.

Isolate	endornaviruses	Mycelial growth inhibition (%) at famoxadone (μg ml $^{-1}$)						
		0.0015	0.015	0.15	1.5	15	150[a]	300[b]
CH98APS060	PEV2&PEV3	0.0	7.0	0.0	22.0**	17.0	84.0	79.6
CH98APS060-a	PEV2&PEV3#	0.0	23.8	73.5	79.6**	78.2	89.0	89.9
CH98ASP059	PEV2&PEV3	0.0	0.0	0.0	15.0**	15.0	72.6	66.6
CH98ASP059-L	PEV2#&PEV3#	0.0	0.0	0.0	0.0**	0.0	0.0	0.0

[a]The recommended concentration for commercial use.
[b]Five times the recommended concentration for commercial use.
** indicate p value < 0.01, respectively (the Tukey-Kramer range test).

Supplementary Table S8. Sensitivities of *Phytophthora* sp. isolates to famoxadone (FA) and/or n-propyl gallate (PG) on potato dextrose agar medium.

Isolate	endornaviruses	Mycelial growth inhibition (%) at		
		FA 1.5μg ml $^{-1}$	FA 1.5μg ml $^{-1}$ + 1mM PG	1mM PG
CH98APS060	PEV2&PEV3	22.0	100.0	59.5
CH98APS060-a	PEV2&PEV3#	79.6	100.0	77.1
CH98ASP059	PEV2&PEV3	15.0	100.0	57.6
CH98ASP059-L	PEV2#&PEV3#	0.0	14.1	25.0
